# Polyploid GWAS reveals the basis of molecular marker development for complex breeding traits including starch content in the storage roots of sweet potato

**DOI:** 10.3389/fpls.2023.1181909

**Published:** 2023-06-05

**Authors:** Emdadul Haque, Kenta Shirasawa, Keisuke Suematsu, Hiroaki Tabuchi, Sachiko Isobe, Masaru Tanaka

**Affiliations:** ^1^ Kyushu Okinawa Agricultural Research Center, National Agriculture and Food Research Organization, Miyakonojo, Japan; ^2^ Department of Frontier Research and Development, Kazusa DNA Research Institute, Kisarazu, Japan

**Keywords:** sweetpotato, complex breeding trait, starch content, SNPs, polyploid GWAS, starch metabolizing gene

## Abstract

Given the importance of prioritizing genome-based breeding of sweet potato to enable the promotion of food and nutritional security for future human societies, here, we aimed to dissect the genetic basis of storage root starch content (SC) when associated with a complex set of breeding traits including dry matter (DM) rate, storage root fresh weight (SRFW), and anthocyanin (AN) content in a mapping population containing purple-fleshed sweet potato. A polyploid genome-wide association study (GWAS) was extensively exploited using 90,222 single-nucleotide polymorphisms (SNPs) obtained from a bi-parental 204 F_1_ population between ‘Konaishin’ (having high SC but no AN) and ‘Akemurasaki’ (having high AN content but moderate SC). Through the comparison of polyploid GWAS on the whole set of the 204 F_1_, 93 high-AN-containing F_1_, and 111 low-AN-containing F_1_ populations, a total of two (consists of six SNPs), two (14 SNPs), four (eight SNPs), and nine (214 SNPs) significantly associated signals were identified for the variations of SC, DM, SRFW, and the relative AN content, respectively. Of them, a novel signal associated with SC, which was most consistent in 2019 and 2020 in both the 204 F_1_ and 111 low-AN-containing F_1_ populations, was identified in homologous group 15. The five SNP markers associated with homologous group 15 could affect SC improvement with a degree of positive effect (~4.33) and screen high-starch-containing lines with higher efficiency (~68%). In a database search of 62 genes involved in starch metabolism, five genes including enzyme genes *granule-bound starch synthase I (IbGBSSI)*, *α-amylase 1D*, *α-amylase 1E*, and *α-amylase 3*, and one transporter gene *ATP/ADP-transporter* were located on homologous group 15. In an extensive qRT-PCR of these genes using the storage roots harvested at 2, 3, and 4 months after field transplantation in 2022, *IbGBSSI*, which encodes the starch synthase isozyme that catalyzes the biosynthesis of amylose molecule, was most consistently elevated during starch accumulation in sweet potato. These results would enhance our understanding of the underlying genetic basis of a complex set of breeding traits in the starchy roots of sweet potato, and the molecular information, particularly for SC, would be a potential platform for molecular marker development for this trait.

## Introduction

Sweet potato [*Ipomoea batatas* (L.) Lam.] is a key crop in Japan (Katayama et al., 2017) and the seventh most important food crop in the world (Food and Agricultural Organization (FAO), 2020). Although young leaves and shoots are sometimes eaten as green vegetables (Kobayashi et al., 2019), sweet potato is mainly bred for its large, starchy, and sweet-tasting storage roots. Many of the important traits related to the productivity of this root organ are quantitatively inherited traits such as storage root yield, dry matter ratio, starch content, and various health-promoting compounds including anthocyanin and carotenoid. Sweet potato cultivars rich in these traits are used as food, feed, nutrition source, and cash crop in developing countries and as food processing component/table use in developed countries including Japan (Katayama et al., 2017). The crop, thus, has a great contribution to humanity. Obviously, the breeding of sweet potato in Japan has surged to develop superior cultivars rich in these traits, and some promising cultivars were developed successfully to address food/nutritional security and industrial needs nationally and internationally (Katayama et al., 2017). In these breeding programs, a cultivar is released through extensive field evaluation of a large number of seedlings in the early stage of breeding and consecutive line selection (Katayama et al., 2017). This is mainly because of the heterozygosity and hexasomic hexaploid nature of sweet potato (2n = 6x = 90), leading to diverse combinations of parental chromosomes in the male and female gametes, which ultimately reduce the chance to obtain a new breeding line in the early cycle of a breeding program. Furthermore, in sweet potato, these agronomic traits accumulate during the developmental process of storage roots (Wang et al., 2016) through either negative or positive association (Yada et al., 2017; Haque et al., 2020a). These associations sometimes make their inheritance complex and, thus, have become the limiting factor when breeding a sweet potato cultivar bearing traits of interest in the same root organ. For example, starch is a basic raw material of sweet potato roots, which accounts for up to 80% of the dry matter (Zhu and Wang, 2014). High starch content with added values such as higher polyphenol content and higher pigment content including anthocyanin is the basic consideration to develop a superior root organ in sweet potato. Therefore, understanding the genetic basis surrounding their association is a prerequisite in a sweet potato breeding program.

Genetic analysis on a trait of interest, i.e., the development of molecular markers, is a long-awaited molecular breeding tool aimed to dramatically reduce the labor effort and breeding period. While the technology is efficient in diploid species, the genetic analysis of sweet potato is complicated by factors associated with heterozygosity and autohexaploidy. Such factors include the presence of multiple alleles at marker loci and differential allele dosages across six homeologous chromosomes, the possibility of both bivalent and multivalent formations during meiosis, and the possibility of preferential pairing during meiosis (Dufresne et al., 2014). The quantitatively inherent nature of most agronomic traits, which are controlled by multiple alleles at multiple loci with high levels of genotype × environment interactions (Yada et al., 2017), further complicated the genetic analysis in this important crop. In the early phase of genetic studies, efforts were made with relatively few amplified fragment length polymorphism (AFLP) and/or simple sequence repeat (SSR) markers (Cervantes-Flores et al., 2011; Nakayama et al., 2012; Zhang et al., 2016; Yada et al., 2017) are not enough to evenly cover the 90 homeologous chromosomes of sweet potato. As next-generation sequencing (NGS)-generated genome-wide single-nucleotide polymorphism (SNP)-based genetic research has progressed (Hirakawa et al., 2015; Shirasawa et al., 2017), we have successfully performed a genome-wide association study (GWAS) and quantitative trait locus (QTL) analysis to identify the responsive SNPs for agronomic traits including β-carotene, dry matter, and starch content (Haque et al., 2020a) or resistance to nematodes (Sasai et al., 2019). We also attempted NGS-generated SNPs coupled with QTL-seq mapping in sweet potato to select strongly associated SNPs followed by the development of tightly linked DNA markers for anthocyanin content (Yamakawa et al., 2021). These studies, however, are still limited to the independent homologous groups and mode of inheritance of Mendelian markers (Shirasawa et al., 2017) or to major gene-regulated traits and can take advantage of only a limited number of co-dominant markers. More versatile co-dominant SNP makers are expected for the development of molecular markers to enable the efficient breeding of polygenic sweet potato traits. More importantly, while DNA markers linked to a responsible gene are expected to be consistent with their respective trait, due to self-infertility, precise mapping and map-based cloning are usually limited to the F_1_ population in cultivated sweet potato.

Recent studies on the integrated pseudochromosomes and polyploid genetic mapping enabling the estimation of multiple-dosage SNP markers in hexasomic hexaploid sweet potato have ushered in a new era in the genetic study of sweet potato (Yoon et al., 2023; Mollinari et al., 2020; Yamamoto et al., 2020). Furthermore, GWAS followed by the analysis of genetic factors targeting the detected SNP loci is an emerging approach being applied as an alternative to map-based cloning in sweet potato. With the use of the polyploid QTL mapping strategy (Mollinari et al., 2020), QTL analysis in dry matter, starch content, and their negatively associated trait β-carotene (Gemenet et al., 2020), was reported in sweet potato. The authors also performed differential gene expression analysis to obtain genetic factors for these agronomic traits. However, to date, no molecular markers that enable efficient selection of these agronomic traits of interest in sweet potato breeding, i.e., marker-assisted selection, have been reported. Furthermore, due to the differences in the background genome sequence, the genetic gains from a given F_1_ population cannot be applied to other F_1_ populations. So far, the genetic studies on starch content, dry matter, storage root yield, and their associated pigment anthocyanin content have not been reported in the same background population, which we termed as a “complex set of breeding traits”. Particularly, the dissection of the genetic basis underlying total starch content in an F_1_ population containing anthocyanin-rich purple-fleshed sweet potato is of great interest.

Linkage-based QTL mapping is mostly performed for bi-parental populations (Cervantes-Flores et al., 2011; Nakayama et al., 2012; Zhang et al., 2016; Sasai et al., 2019; Haque et al., 2020a), mainly due to low-density markers on genetic maps, and for the need to estimate genotype scores at the intervals of two markers, even if the read coverages were insufficient to distinguish three scores, e.g., A, B, and H (Haque et al., 2020b). However, the polyploid GWAS method in sweet potato is based on the physical map of the *Ipomoea trifida* genome, which is available at the chromosome level (Yamamoto et al., 2020). The method does not require genetic maps for representing the genome structure, as the marker density is typically enough to cover the genome. The method has also the merit of error tolerance in genotyping scores and allele dosage probability, calculated from low coverage data and even applicable if the genetic mapping of a polyploid species (bi-parental) could be possible when accurate allele dosage information is not available. The polyploid GWAS method performs a simple marker–phenotype association analysis to detect the genetic loci associated with phenotypic variations (Yamamoto et al., 2020).

Thus, the polyploid GWAS approach is becoming common in hexasomic hexaploid sweet potato (bi-parental) breeding and was found to be effective for mining new genes and molecular markers for a wide number of breeding traits (Haque et al., 2020b; Yamamoto et al., 2020; Obata et al., 2022).

In order to properly elucidate the genetic mechanism underlying anthocyanin accumulation in the storage roots of sweet potato and thereby contribute to the molecular system for anthocyanin differentiation in a sweet potato breeding program, in our laboratory, polyploid GWAS performed with 59,675 SNPs obtained from the 94 F_1_ mapping population between the cultivars ‘Konaishin’ (which has a high yield and starch content but no anthocyanin) and ‘Akemurasaki’ (which has a high anthocyanin content but low-to-moderate yield and starch content) (Haque et al., 2020b). This enabled us to identify 59 genome-wide SNP markers linked to the anthocyanin content. The study was conducted under pot culture conditions in 2018, and out of 59 SNPs, 31 were multiplex (53%) and 28 (47%) were simplex and double-simplex SNPs. In addition, based on the database search results of anthocyanin biosynthesis genes on the associated homologous chromosome using sweet potato genome (Yoon et al., 2023), we clarified the locations of some candidate genes including *IbMYB1*, a transcription factor that was reported to be tightly linked to anthocyanin accumulation in the storage roots (Tanaka et al., 2012). In the undertaken study, a large-scale field study was carried out in 2019 and 2020 using a 204 F_1_ bi-parental population obtained from the same background (Haque et al., 2020b). We measured starch content (SC), dry matter (DM) rate, storage root fresh weight (SRFW), and the relative anthocyanin (AN) content. We applied the polyploid GWAS with 90,222 genome-wide SNPs and reported on the candidate SNP markers for the variations of these traits through an extensive comparison of GWAS results among the 204 F_1_ mapping population, 93 F_1_ population with high AN content, and 111 F_1_ population with low AN content. We also report on the candidate genes within the identified homologous group (HG) that is homologous to the genes associated with the starch metabolism pathway. The objective of this study is to i) understand and dissect the underlying genetic basis of SC in the storage roots of sweet potato when associated with other complex breeding traits and ii) provide a useful resource base for the development of molecular markers for SC.

## Materials and methods

### Plant materials

The mapping population consisting of 204 F_1_ progeny were used from a bi-parental cross between two *I. batatas* cultivars, ‘Konaishin’ (KNS) and ‘Akemurasaki’ (AKM). As described previously (Haque et al., 2020b), the former is a novel cultivar for starch production released in 2018 with no AN content and extraordinarily high yield. However, the latter is a cultivar with significantly high AN content (Sakai et al., 2009), low-to-moderate yield, and moderate SC; 94 F_1_ from this population are used in our previous report (Haque et al., 2020b), and the entire mapping population has been maintained in a greenhouse at Kyushu Okinawa Agricultural Research Center, NARO (KARC/NARO), Miyakonojo, Miyazaki Prefecture, Japan. In the current study, initially, we expected a single mapping population with multiple variable traits including higher SC, basic raw materials of sweet potato root organ, its associated production-related traits DM and yield, and added value trait AN content. Therefore, although the two parents are as contrasting for SC compared to AN content, we have chosen this mapping population for the undertaken study. Furthermore, regarding the 94 F_1_ mapping population, no prior phenotypic selection based on DNA markers has been performed.

### Phenotyping of SRFW, SC, DM, and the relative AN content

The phenotyping trials were carried out for two consecutive years in June to October 2019 and 2020 at KARC/NARO, Miyakonojo, Miyazaki Prefecture, Japan (**Supplementary Figure 1**). Approximately 25–30-cm-long stem cuttings for planting were obtained from the lateral branches nurtured in a polyvinyl house. The experimental field was divided into three-unit quadrats (plots) at 25 m × 12 m each. In each plot, a total of 206 of 1.5-m-long ridged rows were laid out to plant the KNS, AKM, and 204 F_1_ populations. Ridges are spaced 75 cm apart and covered with black polyethylene to maintain temperature and moisture and to inhibit weed growth. On each plot, five cuttings for each genotype were planted spaced 30 cm apart, for a total of 1,030 plants per plot. A cutting of reference genotypes was planted between two genotypes to separate the genotype within the plot. Randomization was performed within the plot. The field trial was under rainfed conditions, with pesticides applied twice and weeded when needed.

Plants were harvested at 4 months after transplantation. After the number and weight (kilograms) of the storage roots were recorded, two to four medium-sized storage roots were selected, and SC and DM were measured as percentages as described previously (Haque et al., 2020a). The relative AN content was analyzed as absorbance at 530 nm (A_530_) according to Haque et al. (2020b).

Pearson’s coefficients of correlation among different traits were calculated using SPSS 19.0 statistical software (SPSS, Chicago, IL, USA). A two-factor ANOVA was performed using RStudio (v1.4.1717-3). Broad-sense heritability was estimated as $h^{2}B=\sigma_{G}^{2}/(\sigma_{G}^{2}+\sigma_{GE}^{2}/e+\sigma_{\epsilon }^{2}/\text{re})$ , where the genetic variance $\sigma_{G}^{2}$ = (MS*_G_* − MS*_GE_*)/*re*, genotype × environment interaction variance $\sigma_{GE}^{2}$ = (MS*_GE_* − MS_ϵ_)/*r*, error variance $\sigma_{\epsilon }^{2}$ = MS*_e_*, MS*_G_* = genotype mean square, MS*_GE_* = genotype × environment interaction mean square, MS*_e_* = error mean square, and *r* and *e* are the numbers of replicates and environments.

**Supplementary Figure 2** shows storage root flesh color in handmade slices.

### Genotyping and polyploid GWAS

The total DNA of each individual and their parents was isolated from folded young leaf tissues. ddRADSeq analysis was performed according to the method of Shirasawa et al. (2016). In total, 93,972,447,270 bases were obtained with HiSeq 4000 system (Illumina, San Diego, CA, USA). After sequence alignment to the *I. trifida* pseudomolecule (Yoon et al., 2023) and filtering, a total of 90,222 SNPs were derived for the 204 F_1_ population using a max missing value of 0.8 (20%). From these SNPs, two subsets of SNPs containing 89,982, and 88,976 sites were derived for the 93 high-AN-containing F_1_ population and 111 low-AN-containing F_1_ populations, respectively.

These SNPs underwent allele dosage estimation according to Yamamoto et al. (2020) with the following criteria: ploidy = 6, minimum read depth (dp) = 10, maximum dp = 1,000, maximum missing = 0.5, maximum frequency = 0.95, round-up = 1.00, cutoff = 0.05, and read error probability = 0.001. Polyploid GWAS was conducted using a generalized linear model according to the method described (Yamamoto et al., 2020). The Manhattan plot was created with “manhattan” in R package *qqman* (Turner, 2014). Regarding the GWAS result, the genome-wide suggestive threshold (suggestive *p*-value value of 1.17 × 10^−5^) was established at −log_10_ *p* > 4.9, based on the previous report (Zhang et al., 2020).

ANOVA, with an aim to filter out any false-positive SNPs, was performed between these SNPs and their associated traits. The stable GWAS signals having at least one significant SNP were declared as candidate GWAS signals.

### Searching the homologous regions of starch-metabolizing genes from other plants in the sweet potato genome

Starch metabolism in higher plants is regulated by enzyme genes, transcription factors, and transporter genes. The majority of them include sucrose synthase, UDP-glucose pyrophosphorylase, beta-fructofuranosidase, ADP-glucose pyrophosphorylases (large subunit and small subunit), starch synthases (the granule-bound starch synthases and the soluble starch synthases), starch-branching enzymes, starch-debranching enzymes (isoamylases and pullulanase), and starch degradation enzymes (α- and β-amylases). Various types and isoforms exist for each type of enzyme in sweet potato (Hirakawa et al., 2015). To identify the homology regions of enzyme genes, transcription factors, and transporter genes associated with the starch metabolism pathway, their cDNAs and genes (when available) (gene ID/accession numbers are listed in **Supplementary Table 2**) were used to query the reference genome database of *I. trifida* (ITR_r2.2. scaffold) (Yoon et al., 2023) using a local blast search (Genetyx ver.15, GENETYX Co.).

### qRT-PCR of genes associated with starch metabolism pathway

Storage roots of KNS, AKM, and their five high- and five low-starch-containing F_1_ lines were grown from June to September 2022 at KARC/NARO under the same experimental field used for GWAS. The planting method, cultivation conditions, and other operations were the same as those of GWAS. Experiments were carried out using a randomized complete block design with three biological replications for each of the three time-point of harvesting (at 2, 3, and 4 months) after field transplantation. A total of 36 samples (12 genotypes with three replications) were collected from each harvest. Thus, in total, 108 root samplings were carried out 2, 3, and 4 months after field transplantation.

Frozen root slices (~2 g) were ground using 10-ml grinding jars (Retsch, Düsseldorf, Germany) containing 10-mm stainless steel balls (AS ONE, Osaka, Japan) and TissueLyser II (Qiagen, Hilden, Germany). Total RNA from finely crushed powder (100~200 mg) was extracted by using an ISOSPIN Plant RNA Kit (NIPPON GENE, Co., Ltd., Tokyo, Japan). cDNA was synthesized from 1 μg of total RNA in a 20-μl reaction volume using a PrimeScript™ RT reagent kit (Takara Bio Inc., Maebashi, Japan). Primer pairs of the respective genes were designed from the conserved regions of KNS (DRR267133) and AKM (DRR267132) (Yamakawa et al., 2021). The nucleotide sequences of the selected five genes and the actin gene (Tanaka et al., 2009) are listed in **Supplementary Table 3**. qRT-PCR was performed with diluted cDNA in a 12.5-μl reaction volume using a CFX Connect Real-Time PCR Detection System (Bio-Rad Laboratories, Hercules, CA, USA) and a TB Green^®^ *Premix Ex Taq™ II* (Tli RNaseH Plus) kit (Takara Bio Inc., Japan) in accordance with the manufacturers’ instructions. The PCR conditions were as follows: 95°C for 30 s, 40 cycles of 95°C for 5 s, 60°C for 30 s, and 72°C for 34 s, and a final melt curve profile (65°C–95°C). The transcript levels of target genes were normalized to *Actin* as an internal control, and the changes in gene expression were calculated relative to AKM plants using the 2^−ΔΔCt^ method.

## Results

### Phenotypic variations in SC, DM, SRFW, and the relative AN content

**Figure 1:**
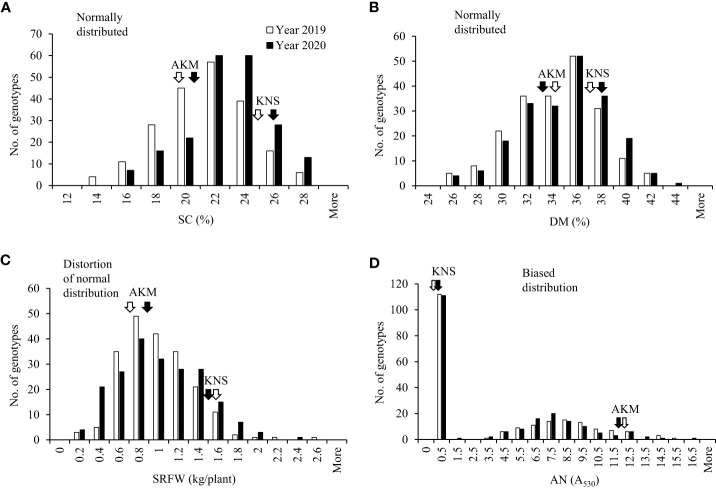
Frequency distribution of the breeding traits in 2019 and 2020 with the F_1_ population between cultivars Akemurasaki (AKM) and Konaishin (KNS). **(A)** SC (%; *n* = 206). **(B)** DM (%; *n* = 206). **(C)** SRFW (kg/plant; *n* = 206). **(D)** AN (A_530_; *n* = 206). Normality was checked with Shapiro–Wilk test at α = 0.05. SC, starch content; DM, dry matter; SRFW, storage root fresh weight; AN, anthocyanin.

The distribution of SC in the storage roots of the 204 F_1_ population was normal (**Figure 1A**). The range of the SC was 13.33%–26.40% (with a population mean of 20.40%) and 14.80%–27.87% (with a population mean of 21.80%) in 2019 and 2020, respectively. The average SC for KNS and AKM was 24.40% and 19.45% in 2019 and 25.77% and 20.83% in 2020, respectively. Transgressive segregation was observed in the 204 F_1_ population (**Figure 1A**). SC showed a strong positive correlation with DM and a weak positive with the relative AN content but no correlation with SRFW (**Table 1**).

**Table 1:** Correlation among traits in the 204 F_1_ population from Konaishin (KNS) and Akemurasaki (AKM).

Trait	Year	SC	DM	SRFW	AN
SC	2019	1			
	2020	1			
DM	2019	0.89^**^	1		
	2020	0.91^**^	1		
SRFW	2019	−0.06	−0.24^**^	1	
	2020	−0.13	−0.25^**^	1	
AN	2019	0.16^*^	0.37^**^	−0.35^**^	1
	2020	0.24^**^	0.45^**^	−0.32^**^	1

SC, starch content; DM, dry matter; SRFW, storage root fresh weight; AN, anthocyanin.

* and ** indicate significant correlation at p< 0.05 and p< 0.01 based on t-test, respectively.

The distribution of DM in the 204 F_1_ population was normal (**Figure 1B**). The range of the DM was 24.07%–41.60% (with a population mean of 33.45%) and 25.47%–42.33% (with a population mean of 33.97%) in 2019 and 2020, respectively. The average DM for KNS and AKM was 37.17% and 34.23% in 2019 and 38.00% and 32.73% in 2020, respectively. Like SC, transgressive segregation was observed for DM in the 204 F_1_ population (**Figure 1B**). DM showed a positive correlation with the relative AN content and a negative correlation with SRFW (**Table 1**).

The distribution of SRFW of the 204 F_1_ population was distorted from a normal distribution (**Figure 1C**). The range of the SRFW was 0.14–2.52 kg/plant (with a population mean of 0.89 kg/plant) and 0.10–2.29 kg/plant (with a population mean of 0.91 kg/plant) in 2019 and 2020, respectively. The average SRFW for KNS and AKM was 1.57 and 0.72 kg/plant in 2019 and 1.47 and 0.88 kg/plant in 2020, respectively. Like other traits, transgressive segregation was observed for SRFW in the 204 F_1_ population (**Figure 1C**). A negative correlation was found between SRFW and the relative AN content (**Table 1**).

The distribution of relative AN content was highly biased, with 54% (*n* = 111) of clones showing a low-to-undetectable level (A_530_< 0.5) (**Figure 1D**). The range of the relative AN content of the high-AN-containing 93 F_1_ population was 3.31–15.43 A_530_ (with a population mean of 8.10 A_530_) and 3.31–15.52 A_530_ (with a population mean of 7.62 A_530_) in 2019 and 2020, respectively. The average relative AN content for KNS and AKM was 0.26 A_530_ and 12.39 A_530_ in 2019 and 0.28 A_530_ and 11.73 A_530_ in 2020, respectively. Transgressive segregation was also observed for the relative AN content (**Figure 1D**).

ANOVA revealed significant genotype-by-year interactions in all the traits studied (**Table 2**). However, high broad-sense heritability was observed for DM (H^2 = ^95.05%), SC (H^2 = ^94.23%), and the relative AN content (H^2 = ^92.00%) compared to SRFW (H^2 = ^75.65), indicating that DM, SC, and the relative AN content are more stably inherited than SRFW (**Table 2**). Similarly, DM (*ρ* = 0.89, *p* = 2.2e−16, *n* = 204), SC (*ρ* = 0.88, *p* = 2.2e−16, *n* = 204), and the relative AN content (*ρ* = 0.83, *p* = 2.2e−16, *n* = 94) showed a strong positive correlation between 2019 and 2020 compared to SRFW (*ρ* = 0.55, *p* = 2.2e−16, *n* = 204) (**Supplementary Figures 3A–D**), indicating that there is higher environmental influence on the F_1_ clones for SRFW than the rest of the traits.

**Table 2:** ANOVA of SC, DM, SRFW, and the relative AN content.

Trait^a^	Variation	DF	SS	MS	F value	Pr > F	*h* ^2^ *B* (%)
SC	Year^b^	1	614	613.6	520.592	<0.001	94.23
	Genotype^c^	205	9,236	45.1	38.227	<0.001	
	Year : Genotype^d^	205	536	2.6	2.22	<0.001	
	Error	822	969	1.2			
DM	Year^b^	1	81	80.61	42.602	<0.001	95.05
	Genotype *^c^*	205	14,119	68.87	36.398	<0.001	
	Year : Genotype^d^	205	699	3.41	1.801	<0.001	
	Error	822	1,555	1.89			
SRFW	Year^b^	1	0.11	0.1108	1.847	0.174	75.65
	Genotype *^c^*	205	149.03	0.727	12.119	<0.001	
	Year : Genotype^d^	205	36.31	0.1771	2.953	<0.001	
	Error	822	49.31	0.06			
AN	Year^b^	1	29	29.00	24.076	<0.001	92.00
	Genotype *^c^*	93	3374	36.28	30.116	<0.001	
	Year : Genotype^d^	93	270	2.90	2.409	<0.001	
	Error	822	462	0.56			

SC, starch content; DM, dry matter; SRFW, storage root fresh weight; AN, anthocyanin; h^2^B, broad-sense heritability; DF, degrees of freedom; SS, sum of squares; MS, mean sum of squares (SS/DF).

a While 206 individuals (204 F_1_ population and parents) were used for SC, DM, and SRFW, 94 individuals (93 high-AN-containing F_1_ population and parent AKM) were used for the relative AN content.

b Mean squares test the significant effect of years 2019 and 2020.

c Test the significant effect of least significant means of individual genotypes (parents and progeny) across seasons.

d Test the significant effect of genotype × environment interaction.

Since the segregation ratio of the high-AN-containing population (*n* = 93) and low-AN-containing population (*n* = 111) fits an expected segregation ratio of 1:1 (*χ* ^2 = ^1.417, *p* = 0.234) (**Supplementary Figure 2**), the effect of AN on the variations of other traits was investigated on both the data sets. While the SRFW of the low-AN-containing F_1_ population was significantly higher than that of the high-AN-containing F_1_ population (**Supplementary Figure 4C**), the scenario for SC and DM was inverse (**Supplementary Figures 4A, B**). In histogram analysis of these two categories, SC, DM, and SRFW (**Supplementary Figures 5A–C**) showed a similar distribution pattern as observed in the 204 F_1_ population (**Figure 1**), except for SRFW in the high-AN-containing population with normal distribution instead of distortion from a normal distribution (**Supplementary Figure 5C**, top panel). We also investigated the effect of β-carotene content (A_455_; Haque et al., 2020a), which appeared in a number of F_1_ genotypes (**Supplementary Figure 2**), on SC and the rest of the traits using the 94 F_1_ population derived from KNS × AKM; however, no or very weak correlation was found (*data not shown*). Taken together, the whole set of the 204 F_1_ (designated as WSF_1_) population, high-AN-containing 93 F_1_ (HAF_1_) population, and low-AN-containing 111 F_1_ (LAF_1_) population was used to search for GWAS. Significant loci and associated SNPs were declared when they were significant in at least one respective year.

### GWAS of SC, DM, SRFW, and the relative AN content using WSF_1_, HAF_1_, and LAF_1_ populations

**Figure 2:**
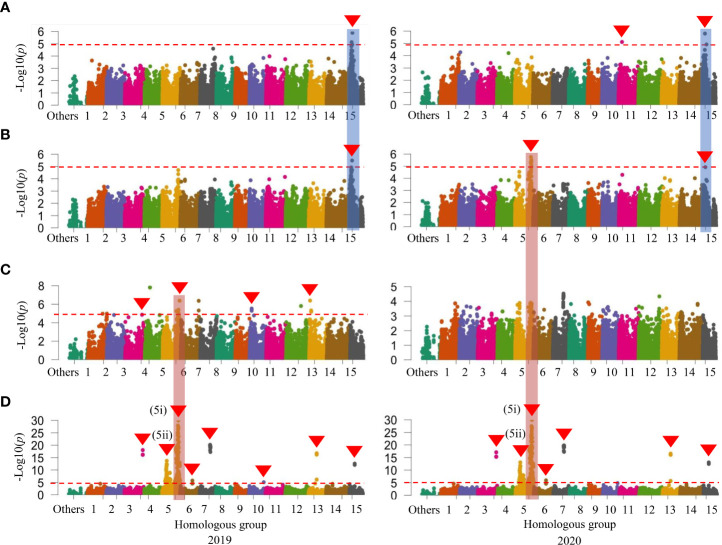
Manhattan plots in the whole set of 204 F_1_ (designated as WSF_1_) population. **(A)** SC (%). **(B)** DM (%). **(C)** SRFW (kg/plant). **(D)** AN. The horizontal dashed red line represents the significance thresholds. The arrowhead indicates the location of the signal having significant SNP. Peaks that appeared in a similar position on the chromosome between or among traits are also marked with vertical shadows. SC, starch content; DM, dry matter; SRFW, storage root fresh weight; AN, anthocyanin; SNP, single-nucleotide polymorphism.

**Figure 3:**
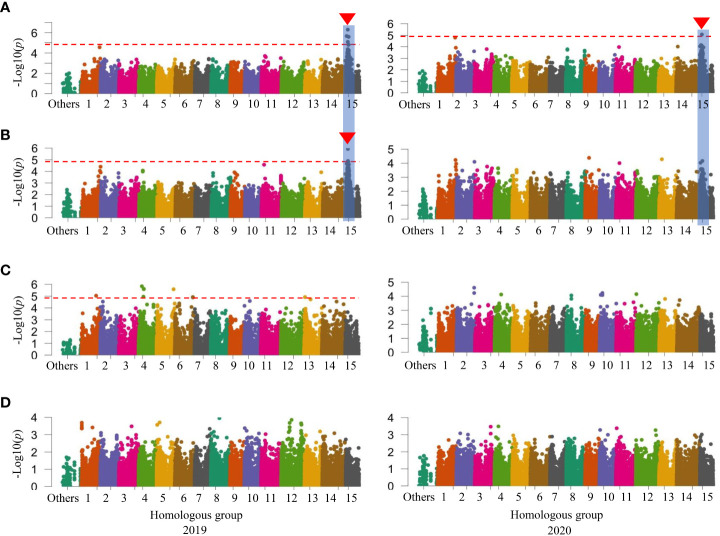
Manhattan plots in the low-AN-containing 111 F_1_ (LAF_1_) population. **(A)** SC (%). **(B)** DM (%). **(C)** SRFW (kg/plant). **(D)** AN. The horizontal dashed red line represents the significance thresholds. The arrowhead indicates the location of the signal having significant SNP. Peaks that appeared in a similar position on the chromosome between or among traits are also marked with vertical shadows. AN, anthocyanin; SC, starch content; DM, dry matter; SRFW, storage root fresh weight; SNP, single-nucleotide polymorphism.

When investigated GWAS in all three sets of mapping populations in 2019 and 2020, a signal on HG 15 associated with SC was initially identified in the WSF_1_ population (**Figure 2A**) and became more prominent in the LAF_1_ population (**Figure 3A**), while a second signal was also identified on HG 11 in the WSF_1_ population only in 2020 (**Figure 2A**). As shown in **Table 3**, on HG 15, three significant SNPs (*p* = 1.17 × 10^−05^–1.58 × 10^−06^) were associated with the WSF_1_ population and four SNPs (*p* = 8.48 × 10^−06^–5.07 × 10^−07^), including two of the above three SNPs, in the LAF_1_ population. Only one SNP (*p* = 7.65 × 10^−06^) was identified on HG 11. GWAS for the variation of DM, greatly followed by SC, identified one major signal on HG 15 in the WSF_1_ population (**Figure 2B**) and/or LAF_1_ population (**Figure 3B**). The signal consisted of two significant SNPs (*p* = 1.18 × 10^−05^ and *p* = 3.24 × 10^−06^) in the WSF_1_ population and four SNPs (*p* = 1.18 × 10^−05^–4.27 × 10^−06^) in the LAF_1_ population, including one of the two identified in the WSF_1_ population. With respect to the signal on HG 15, two SNPs (ITR_CHR15_2753578 and ITR_CHR15_4116419) were commonly identified between these two traits. A second significant signal for the variation of DM was identified on HG 5 of the WSF_1_ population only in 2020. A total of nine SNPs were identified on HG 5 for the variation of DM. All nine and one of them were commonly identified in the same region for the variations of the relative AN content and SRFW, respectively.

**Table 3:** Summary of the SNP markers for the variations of SC, DM, and SRFW.

Trait	Population	Marker^a^	HG^b^	Year	*p*-Value (2019/2020)	ANV^c^ (2019/2020)	MD^d^ (2019/2020)	
SC	WSF_1_	ITR_CHR11_5482159	11	2020	7.65 × 10^−06^	*	0.90	
		**ITR_CHR15_2753578^dm^**	15	2019	7.37 × 10^−06^	**	1.20	
		ITR_CHR15_3884083	15	2019	1.17 × 10^−05^	***	1.51	
		**ITR_CHR15_4116419^dm^**	15	2019/2020	2.59 × 10^−06^/1.58 × 10^−06^	**/**	1.13/1.15	
	HAF_1_	NA						
	LAF_1_	ITR_CHR15_985591	15	2019	2.17 × 10^−06^	***	4.33	
		ITR_CHR15_2753575	15	2019	8.03 × 10^−06^	***	1.96	
		**ITR_CHR15_2753578^dm^**	15	2019	5.07 × 10^−07^	**	1.68	
		**ITR_CHR15_4116419^dm^**	15	2019/2020	2.58 × 10^−06^/8.48 × 10^−06^	*/*	1.30/1.41	
DM	WSF_1_	ITR_CHR05_26029224^an^	5	2020	5.17 × 10^−06^	***	2.28	
		ITR_CHR05_26396741^an&srfw^	5	2020	1.74 × 10^−06^	***	1.63	
		ITR_CHR05_26701979^an^	5	2020	1.20 × 10^−05^	**	1.61	
		ITR_CHR05_26718670^an^	5	2020	1.37 × 10^−05^	***	2.12	
		ITR_CHR05_27046380^an^	5	2020	4.08 × 10^−06^	***	2.45	
		ITR_CHR05_27046381^an^	5	2020	2.96 × 10^−06^	***	2.51	
		ITR_CHR05_27046400^an^	5	2020	3.80 × 10^−06^	***	2.46	
		ITR_CHR05_27046418^an^	5	2020	4.72 × 10^−06^	***	2.59	
		ITR_CHR05_27090704^an^	5	2020	8.59 × 10^−06^	***	1.67	
		**ITR_CHR15_2753578^sc^**	15	2019	1.18 × 10^−05^	**	1.42	
		ITR_CHR15_4116419^sc^	15	2019/2020	3.24 × 10^−06^/1.20 × 10^−05^	**/**	1.35/1.32	
	HAF_1_	NA						
	LAF_1_	I**TR_CHR15_2753578^sc^**	15	2019	1.16 × 10^−06^	**	1.79	
		ITR_CHR15_2874647	15	2019	1.26 × 10^−05^	***	2.50	
		ITR_CHR15_2874901	15	2019	1.38 × 10^−05^	***	2.50	
		ITR_CHR15_2874934	15	2019	1.39 × 10^−05^	***	2.53	
SRFW	WSF_1_	ITR_CHR03_28126752	3	2019	1.37 × 10^−05^	*	−0.14	
		ITR_CHR05_22832191^an^	5	2019	5.55 × 10^−06^	***	−0.27	
		ITR_CHR05_26351002^an^	5	2019	8.65 × 10^−06^	***	−0.24	
		ITR_CHR05_26396741^an&dm^	5	2019	3.95 × 10^−06^	**	−0.16	
		ITR_CHR10_3071150	10	2019	3.96 × 10^−06^	*	0.11	
		ITR_CHR10_3071152	10	2019	2.95 × 10^−06^	*	0.11	
		ITR_CHR10_3071311	10	2019	5.04 × 10^−06^	*	0.11	
		ITR_CHR13_1142241	13	2019	4.02 × 10^−07^	*	0.10	
	HAF_1_	NA					
	LAF_1_	NA					

SNP, single-nucleotide polymorphism; SC, starch content; DM, dry matter; SRFW, storage root fresh weight.

a Marker order was sorted by position on the HG. ITR indicates *Ipomoea trifida*, CHR indicates the chromosome, and the numbers indicate the position (bp) of each marker on its respective chromosome. Bold SNPs were common among populations of each trait. SNPs that were common between or among traits were indicated with trait names.

b HG indicates homologous group.

c ANV indicates ANOVA. *, ** and *** indicate significant differences between genotypes by ANOVA at p< 0.05, p< 0.01, and p< 0.001, respectively.

d MD indicates the mean differences of SC, DM, and SRFW of the F_1_ populations according to the homozygous and heterozygous genotypes of the SNP markers.

For the variation of SRFW, GWAS identified four significant signals including eight SNPs on HGs 3, 5, 10, and 13 in the WSF_1_ population (**Figure 2C**). All three SNPs identified on HG 5 were commonly identified in the same region in the WSF_1_ population for the variation of the relative AN content (**Figure 2D**). In the WSF_1_ population, GWAS identified eight significant signals on HGs 3, 5 (two signals), 6, 7, 10, 13, and 15 for the variation of the relative AN content (**Figure 2D**), while there was only one signal on HG 9 in the HAF_1_ population (**Supplementary Figure 6D**). These signals consisted of a total of 214 SNPs (**Supplementary Table 1**), out of which 208 were common in both years. Furthermore, these SNPs reproduced all the 59 SNPs as reported previously (Haque et al., 2020b).

**Figure 4:**
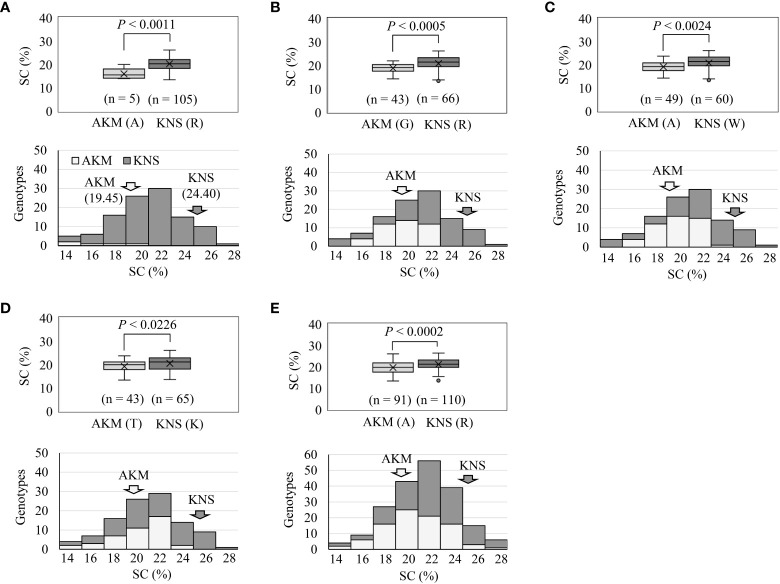
Important features of the SNP markers from HG 15 toward molecular marker development for the high SC (%). Top panels of **(A–E)** indicate the markers ITR_CHR15_985591, ITR_CHR15_2753575, ITR_CHR15_2753578, and ITR_CHR15_4116419 in LAF_1_ mapping population from 2019 and the marker ITR_CHR15_3884083 in WSF_1_ mapping population from 2019. Based on the genotypes of SNPs, the average SC (%) scores of F_1_ lines are plotted. *p*-Value is significant based on one-way ANOVA. Bottom panels of **(A–E)** indicate frequency distribution of the SC (%) grouped by genotypes, based on SNP marker. Marker order is the same as the above. The putative effects of each SNP marker to screen high-starch-containing lines were determined as the percentage of [={the number of high-starch-containing (>20%) KNS lines + the number of low-starch-containing (<20%) AKM lines}/total number of F_1_ lines]. AKM, Akemurasaki; KNS, Konaishin; SNP, single-nucleotide polymorphism; SC, starch content; DM, dry matter; SRFW, storage root fresh weight; AN, anthocyanin.

Overall, the comparative polyploid GWAS on WSF_1_, LAF_1_, and HAF_1_ populations revealed the most stable and novel QTL in this study for SC (followed by DM) on HG 15. To evaluate the effects of the peak observed on HG 15 for the variation of SC, the F_1_ plants were grouped based on the genotypes (homozygous or heterozygous) of the identified SNPs (**Table 3**). With an increasing effect on SC, the five SNP markers on HG 15 exhibited mean differences of 1.13–4.33. The SNP marker ITR_CHR15_985591 showed the highest increasing effect on SC with a significant mean difference of 4.33 (*p*< 0.001) (**Table 3**). These five SNP markers covered approximately 3.31 Mbp of the GWAS signal on HG 15 (**Supplementary Figure 7**). In order to gain insight into the molecular marker development for the high SC (%) in sweet potato, important features of these SNPs markers were also investigated (**Figure 4**). As expected, when plotted based on the genotypes of these SNPs, the lines with the KNS genotypes indicate high SC (top panels of **Figures 4A–E**). The *χ* ^2^ goodness-of-fit test suggested that ITR_CHR15_985591, ITR_CHR15_2753578, and ITR_CHR15_3884083 are triplex, simplex, and simplex dosages of markers, respectively; the ITR_CHR15_2753575 and ITR_CHR15_4116419 markers nearly fit to 1:1 (simplex markers). As shown in the bottom panels of A–E, markers ITR_CHR15_985591, ITR_CHR15_2753575, ITR_CHR15_2753578, ITR_CHR15_4116419, and ITR_CHR15_3884083 showed 55%, 68%, 64%, 53%, and 63% putative efficiencies to screen high-starch-containing F_1_ lines, respectively.

### Candidate genes on HG 15 associated with starch metabolism pathway

In total, 57, 4, and one sequences were used in database search from seven, four, and one of the enzyme genes, transcription factors, and transporter genes, respectively (**Supplementary Table 2**). We found that the five genes, four from two types of enzyme genes most homologous to *granule-bound starch synthase I* (*IbGBSSI*), *α-amylase 1D* (*ItAMY1D*) (also located on HG 4), *α-amylase 1E* (*ItAMY1E*) (also located on HG 4), and *α-amylase 3* (*ItAMY3*) (also located on HG 10) and one transporter gene homologous to *ATP/ADP-transporter* (*StAATP*) (also located on HG 9) were located on HG 15 of the *I. trifida* pseudomolecule (**Table 4**).

**Table 4:** List of candidate genes located on HG 15 associated with starch metabolism pathway.

Name^a^	Gene ID/accession no.	HG^b^ (*Ipomoea trifida*)	Position within *I. trifida^c^*
*Granule-bound starch synthase I (IbGBSSI)*	AB071604	15	5991269-5994843
*α-Amylase 1D (ItAMY1D)*	Itr_sc000553.1_g00024.1	15	3190125-3192261
		4	2822108-2830492
*α-Amylase 1E (ItAMY1E)*	Itr_sc000553.1_g00023.1	15	3193662-3195458
		4	2818651-2823114
*α-Amylase 3 (ItAMY3)*	Itr_sc000559.1_g00005.1	10	932228-8935369
		15	20494944-20501917
*ATP/ADP-transporter (StAATP)*	Y10821	15	318247-321635
		9	6034762-6037927

a Homology searches against ITR r2.2 were performed with BLAST program using the above sequences as queries.

b HG indicates homologous group.

c Homologous position covering all the matched regions with high sequence identity in ITR r2.2 genome.

The positions of these five candidate genes were predicted on HG 15 (**Supplementary Figure 7B**). *ItAMY1D*, *ItAMY1E*, and *IbGBSSI* seem to be within or adjacent to the detected SNP loci, while *ItAMY3* and *StAATP* were a bit far from the SNP loci.

### Expressions of starch-metabolizing genes from HG 15 in the field-grown storage roots of high- and low-starch-containing plants

**Figure 5:**
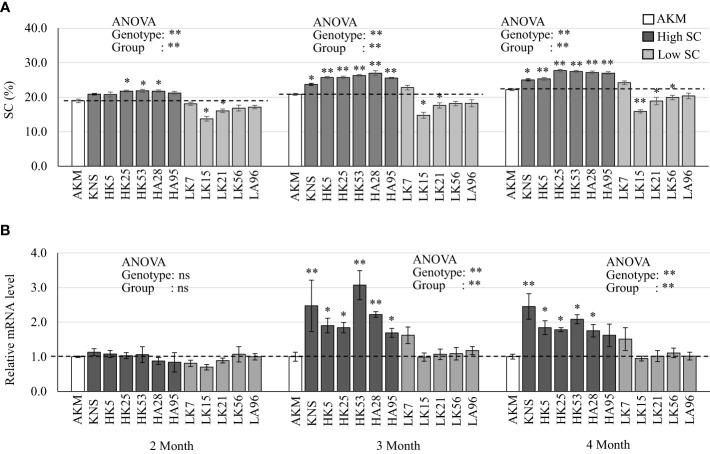
Real-time PCR of starch-metabolizing genes in the storage roots of AKM, KNS, five high-starch-containing F_1_, and five low-starch-containing F_1_ lines at 2, 3, and 4 months after field transplantation. **(A)** SC (%). Single and double asterisks above the bars indicate statistically significant differences at *p*< 0.05 and *p*< 0.01 based on the pairwise *t-*test, respectively. **(B)** The expression of *IbGBSSI*. Gene expressions are presented relative to the expression level of AKM plants. The error bars represent the standard error of the measurement for three independent biological replications (*n* = 3). Transcript levels were normalized to an *IbActin* gene as an internal control. Asterisks above the bars indicate ΔCt values [=Ct(*target*) − Ct (*Actin*)] that are significantly different from those of the AKM plants, as revealed by pairwise *t*-test (*p< 0.05; **p< 0.01). For analysis of variance (ANOVA) among 12 genotypes or three groups (AKM, high SC, and low SC), ns, single, and double asterisks indicate non-significance, *p*< 0.05, and *p*< 0.01, respectively. H, high SC-containing F_1_ lines; L, low-starch-containing F_1_ lines; AKM, Akemurasaki; KNS, Konaishin; SC, starch content; DM, dry matter; SRFW, storage root fresh weight; AN, anthocyanin.

In order to link with the starch accumulation during storage root development, the expression patterns of five candidate genes (*IbGBSSI*, *IbAMY1D*, *IbAMY1E*, *IbAMY3*, and *AATP*) from HG 15 (**Table 4**) were extensively studied on the field-grown storage roots of KNS, AKM, and their five high-starch-containing and five low-starch-containing F_1_ lines. Variations in SC were observed among 12 genotypes as well as three groups (AKM, high-containing, and low SC-containing progeny), which were prominent from 3 months after field transplantation (**Figure 5A**). When compared with that of the AKM, the average SC in the storage roots of KNS and five high-starch-containing groups was greatly increased from 2 months (10.0%–15.0%) to 3 months (14.0%–30.0%) after field transplantation and then was stable up to 4 months (12.0%–24.0%) (**Figure 5A**). As expected, qRT-PCR of *IbGBSSI* expression showed wide variations among genotypes as well as groups from 3 months after field transplantation (**Figure 5B**). The *IbGBSSI* in KNS and high SC-containing group showed considerable consistency, with the SC exhibiting an average of 1.7- to 3.1-fold higher expression at 3 months than that of AKM plants. In contrast, the expression of *IbAMY1D* (**Supplementary Figure 8A**) and *IbAMY1E* (**Supplementary Figure 8B**) did not show remarkable alteration, exhibiting an overall suppression by both the high- and low-starch-containing groups except an unstable upregulation by low-starch-containing groups at 2 months. The mRNA expression of *IbAMY3* (**Supplementary Figure 8C**) and *IbAATP* (**Supplementary Figure 8D**) were not significantly altered in this study.

## Discussion

In Japan, the genome breeding method is the top priority to facilitate sweet potato breeding of important traits with a sufficient amount of background SC. Here, the comparative polyploid GWAS on the WSF_1_, HAF_1_, and LAF_1_ populations enabled us to identify multiple candidate SNP DNA markers linked to SC, DM, SRFW, and the relative AN content, a complex set of breeding traits in sweet potato root organ, and thereby inheritance on their respective HGs. Specifically, the five SNP markers on the most stable and novel signal of HG 15, which presents simple and triple dosages, could affect SC improvement with a degree of positive effect up to 4.33 (**Table 3**) and were assumed to screen high-starch-containing lines with higher efficiency (~68%) (**Figure 4**). The SNP locus may contain genes controlling SC and thus would be a step toward the basis of molecular marker development for this trait.

### Unraveling the inheritance pattern and candidate SNPs for complex root traits

The highly positive correlation between DM and SC found in this study (**Table 1**) can be confirmed by our findings that the putative GWAS signal on HG 15 for SC was also mapped to the same region for DM in the WSF_1_ and LAF_1_ populations where two associated SNPs were common between them (**Table 3**). On HG 15, all the SNP loci for SC and DM exhibited by the WSF_1_ and LAF_1_ populations had a positive effect on the variation of these two traits (**Figures 2**, **3**; **Table 3**). These results agreed with the fact that their inheritance was derived from KNS, which has higher SC and DM (**Figures 1A, B**). The positive-effect SNPs for the variation of DM, which were identified only on HG 5 of the WSF_1_ population in 2020 but not in the LAF_1_ population, were also mapped to the same region for the relative AN content. This can be confirmed by the positive correlation between DM and AN (**Table 1**).

The positive-effect SNPs of SRFW on HGs 10 and 13 of the WSF_1_ population (**Figure 2C**; **Table 3**) suggest that they were inherited from KNS, which has higher SRFW. The SNPs, which were associated with HGs 3 and 5 of the WSF_1_ population, had negative effects on the variation of SRFW (**Figure 2C**; **Table 3**) and suggest that they were inherited from AKM, which has low SRFW (**Figure 1C**). These SNP regions should suppress the SRFW phenotype in AKM, resulting in low SRFW (**Figure 1C**), as previously reported in such kinds of SNPs for other traits in sweet potato (Haque et al., 2020a). Interestingly, the SNPs from HG 5 were also mapped for DM and the relative AN content in the WSF_1_ population (**Table 3**). This can be explained by the negative correlation of SRFW with DM and the relative AN content (**Table 1**). For the variation of relative AN content, all the SNPs that were identified in the WSF_1_ and HAF_1_ populations (**Figure 2D**; **Supplementary Figure 6D**; **Supplementary Table 1**) had a positive effect and suggests that they were inherited from AKM, which has a higher AN content. The identified locus for the relative AN content found in this study coincided with the locus reported in the previous papers (Zhang et al., 2020; Yamakawa et al., 2021). In these reports, the locus for the relative AN content was detected in Chr 12 of the reference sequence of *I. trifida* (NCNSP0306), which corresponds to Chr 5 of ITR_r2.2.

For the variation of SRFW, no GWAS signal and associated SNPs were identified in the LAF_1_ population (**Figure 3C**; **Table 3**). For the variation of the relative AN content, not only one SNP on HG 9 in the HAF_1_ population (**Supplementary Figure 6D**) but also a number of GWAS signals and associated SNPs of the WSF_1_ population (**Figure 2D**; **Supplementary Table 1**) were expected to be reproduced by the HAF_1_ population. At present, we do not know the precise reason. The lower range of genetic variations for SRFW in the LAF_1_ population (0.30–2.29 kg/plant) (**Supplementary Figure 5C**, bottom panel) compared to that of the WSF_1_ population (0.10–2.52 kg/plant) (**Figure 1C**) or the lower range of genetic variations for the relative AN content in the HAF_1_ population (3.31–15.52 A_530_) (**Supplementary Figure 5D**, top panel) compared to that of the WSF_1_ population (0.11–15.52 A_530_) (**Figure 1D**) cannot be ignored. Because KNS is the donor of SRFW, DM, and SC, in this study, higher average SRFW observed in the LAF_1_ population than that in the HAF_1_ population (**Supplementary Figure 4C**) is expected; however, the average SC (**Supplementary Figure 4A**) and DM (**Supplementary Figure 4B**) were contrastingly higher in the HAF_1_ population than those in the LAF_1_ population. The relative AN content is a negative correlator of SRFW, suggesting that genotypes containing lower levels of AN content are more likely to have higher levels of SRFW, i.e., to have larger storage roots. The scenario is inverse for SC or DM. The genotypes containing the higher levels of the relative AN content had narrower storage roots, i.e., higher levels of SC and therefore higher DM. Taken together, SC, DM, SRFW, and the relative AN content are a complex set of breeding traits to be considered in a purple-fleshed single mapping population and need to be confirmed in future studies.

### Understanding the candidate gene(s) homologous to starch metabolism pathway

Starch is solely made up of glucose residues linked by α-(1→4) and α-(1→6) glycosidic linkages. As a biomacromolecule, starch consists of two major polymers; linear and slightly branched amylose (20%–30% of total starch) and highly branched amylopectin (70%–80% of total starch), with approximately 5% α-(1→6) branch points (Wu et al., 2014; Lai et al., 2016). The metabolism of starch granules requires the concerted actions of various types and isoforms of enzymes, transcription factors, and transporter genes. The pathway from ADP-glucose is catalyzed by multiple enzymes (Kitahara et al., 2007). This serves as the substrate for starch synthases, which catalyze the elongation of glucan chains of α-(1→4)-linked glucose residues from the non-reducing end. By interpreting the elevated gene expression results of qRT-PCR in the starch-containing storage roots, we tried to clarify the key genetic factor(s) in which five genes including enzyme genes *IbGBSSI*, *AMY1D*, *AMY1E*, and *AMY3* and one transporter gene *AATP* were found to be located on HG 15 (**Table 4**).

*GBSSI* has been well characterized in other plant species as well as in sweet potato (Zeeman et al., 2010). Suppression of *IbGBSSI* by co-suppression or RNA interference results in the production of amylose-free starch in sweet potato storage roots (Kimura et al., 2001; Kitahara et al., 2007; Otani et al., 2007). Otani et al. (2007) reported that the SC of the storage roots of amylose-free transgenic plants was lower than that of non-transgenic wild type. Recently, CRISPR/Cas9-based knocked out of *IbGBSSI* in sweet potato significantly reduced the amylose percentage in the storage roots (Wang et al., 2019). Although the total starch contents were not changed up to a significant level, the knocked-out lines showed a lower amount in SC. Compared to other SS isoforms, GBSS is a low-copy gene predominantly present in the storage roots of sweet potato (Kimura et al., 2000). The higher expression tendency of *IbGBSSI* (**Figure 5B**) in KNS and high-starch-containing F_1_ lines consistent with SC (**Figure 5A**) suggest that *IbGBSSI* is one of the genetic factors involved in the storage roots of white-fleshed LAF_1_ plants (**Figure 1**; **Supplementary Figure 2**). In this study, LK7 from the low-starch-containing F_1_ lines showed moderately high SC as well as a higher level of *IbGBSSI* expression from 3 months after field transplantation. Although it is unexpected, one of the reasons might be the environmental influence between the GWAS years (2019 or 2020) and the qRT-PCR year (2022). The year-to-year correlation coefficient in sweet potatoes for agronomic traits including SC was reported to be greatly affected by the genetic variation in the population and the environmental factor (Yoshida, 1985). It is noteworthy that out of the five low-starch-containing F_1_ lines, LK7 was selected as a fifth-ranked plant having low-to-moderate SC over GWAS year (15.90% in 2019 and 18.33% in 2020). AMY (EC 3.2.1.1) is an endoamylase hydrolyzing α-(1→4) linkages to release soluble glucans, either linear or branched (Wu et al., 2014). AMY is known to be the main enzyme involved in the degradation of storage starch granules. One of the hypotheses on the lower expression of the two α-amylases *IbAMY1D* (**Supplementary Figure 8A**) and *IbAMY1E* (**Supplementary Figure 8B**) in KNS and high-starch-containing F_1_ lines at 2 months is that these genes might play some sort of role during sweet potato storage root development by lowering the rate of starch degradation. However, the overall suppression of these two genes not only in high-starch-containing lines but also in low-starch-containing lines suggests that these two AMY genes have low expression in the storage roots. No alteration in the expression of another α-amylase *IbAMY3* (**Supplementary Figure 8C**) between high-starch-containing and low-starch-containing F_1_ lines suggests that this gene might equally contribute to the starch accumulation in these two groups (**Supplementary Figure 8C**). AATP, a plastidic ATP/ADP transporter protein, imports ATP from the cytosol into plastids, resulting in an increase in the ATP supply to facilitate anabolic processes such as starch and fatty acid synthesis (Möhlmann et al., 1998). Thus, AATP is not pathway specific, and in our qPCR analysis, *AATP* was unresponsive in the storage roots of high-starch-containing lines compared to low-starch-containing lines (**Supplementary Figure 8D**).

**Figure 6:**
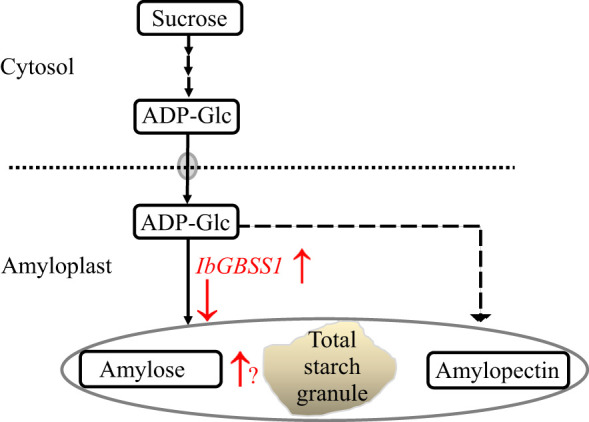
The action positions (red color) of *IbGBSSI* in a proposed model of starch accumulation. Starch metabolism pathway is shown by solid arrows. “↑” and “↓” indicate up- and down-regulation of the relative substrate or enzyme encoding genes.

In light of the above discussion, we postulated that *IbGBSSI* is most probably harbored by HG 15 as one of the candidate genes in determining SC in the storage roots of sweet potato mainly *via* the enhancement of amylose (**Figure 6**). Further study is necessary not only to clarify the above hypothesis but also to know the precise mechanism of *IbGBSSI* gene in starch accumulation using a new mapping population or by means of overexpressed and/or knockdown plants.

In conclusion, we claimed that candidate SNP DNA markers detected with polyploid GWAS for the variation of a set of complex breeding traits in a single mapping population of sweet potato are informative, which is expected to enhance our understanding of mechanisms underlying their inheritance. With special importance on SC, one of the five SNP markers from HG 15 is assumed to include *IbGBSSI* as a candidate gene that might act as a major factor in determining SC in the storage roots of sweet potato. However, in this study, the analysis was performed using a moderate-size bi-parental F_1_ population and not a diverse set of germplasm; i.e., the study has not been validated in different backgrounds. Therefore, in the future study in our laboratory, we aimed to perform GWAS using a greater number of F_1_ mapping populations and/or other F_1_ mapping populations and various sweet potato cultivars/lines to validate and extend the application of this study. Overall, this study provides a model for identifying genomic regions and responsible candidate gene *IbGBSSI* strongly associated with starch accumulation, which have not been reported in sweet potato storage roots to our knowledge. The results not only provide the potential platform for molecular marker development of this trait but also would resolve conflicting findings when breeders consider developing a superior root organ bearing enough background starch content with added values including higher anthocyanin content.

## Data availability statement

The datasets presented in this study can be found in online repositories. The names of the repository/repositories and accession number(s) can be found below: DNA Data Bank of Japan (DDBJ) accession number: DRA015713.

## Author contributions

MT conceived and supervised the research. MT and EH developed the F_1_ mapping population. EH performed the experiments. EH, HT, and MT obtained the phenotypic data. KSh and SI performed ddRADSeq analyses. MT and EH performed polyploid GWAS. EH and KSu performed qRT-PCR. EH performed data analysis and wrote the manuscript. All authors contributed to the article and approved the submitted version.
