# Supplementary material for: Polyploid GWAS reveals the basis of molecular marker development for complex breeding traits including starch content in the storage roots of sweet potato
Source: Front Plant Sci. 2023 Jun 5;14:1181909. doi: 10.3389/fpls.2023.1181909 (PMC10277646; doi:10.3389/fpls.2023.1181909)
Supplement: Supplementary file 1 [file DataSheet_1.pdf]

Supplementary Material

Supplementary Table 1. Summary of the responsive SNP markers for the relative anthocyanin (AN) content above the *qqman* significance threshold.

Population	Marker[a]	HG[b]	Year[c]	*p*-value (2019/2020)	ANOVA[d] (2019/2020)	MD[e] (2019/2020)
WSF_1	**ITR_CHR03_30274266**	**3**	**2019, 2020**	**9.15×10^{-19} /8.01×10^{-18}**	*****/*****	**5.51/4.95**
	ITR_CHR03_30274404	3	2019, 2020	6.40×10^{-17} /4.49×10^{-16}	***/***	5.23/4.67
	ITR_CHR03_30274406	3	2019, 2020	6.87×10^{-17} /4.69×10^{-16}	***/***	5.02/4.50
	ITR_CHR05_4968111	5	2019, 2020	2.39×10^{-06} /7.71×10^{-06}	***/***	3.56/3.28
	ITR_CHR05_6064788	5	2019, 2020	3.15×10^{-07} /7.63×10^{-07}	***/***	3.05/2.76
	ITR_CHR05_6461921	5	2019, 2020	4.09×10^{-07} /3.34×10^{-07}	**/**	1.99/1.92
	ITR_CHR05_6462010	5	2019, 2020	1.87×10^{-07} /2.18×10^{-07}	***/***	2.55/2.32
	ITR_CHR05_6709114	5	2019, 2020	5.64×10^{-11} /1.88×10^{-10}	***/***	3.49/3.30
	ITR_CHR05_6709149	5	2019, 2020	6.14×10^{-11} /1.05×10^{-10}	***/***	3.34/3.15
	ITR_CHR05_6989978	5	2019, 2020	1.96×10^{-10} /8.57×10^{-10}	***/***	4.42/3.96
	ITR_CHR05_7033365	5	2019, 2020	3.39×10^{-11} /9.64×10^{-11}	***/***	3.66/3.42
	ITR_CHR05_7134260	5	2019, 2020	4.65×10^{-12} /2.04×10^{-11}	***/***	3.52/3.13
	ITR_CHR05_7167244	5	2019, 2020	3.04×10^{-07} /2.40×10^{-07}	***/***	3.21/2.93
	ITR_CHR05_7176941	5	2019, 2020	3.87×10^{-08} /1.31×10^{-07}	***/***	3.15/2.76
	ITR_CHR05_7178006	5	2019, 2020	6.02×10^{-09} /4.07×10^{-08}	***/***	3.37/3.00
	ITR_CHR05_7197644	5	2019, 2020	1.03×10^{-07} /2.90×10^{-08}	***/***	3.59/3.38
	ITR_CHR05_7293106	5	2019, 2020	7.02×10^{-08} /6.51×10^{-08}	***/***	2.86/2.65

Supplementary Table 1. Summary of the responsive SNP markers for the relative anthocyanin (AN) content above the *qqman* significance threshold (continue).

Population	Marker[a]	HG[b]	Year[c]	*p*-value (2019/2020)	ANOVA[d] (2019/2020)	MD[e] (2019/2020)
WSF_1	ITR_CHR05_7350280	5	2019, 2020	4.08×10^{-12} /2.20×10^{-11}	***/***	3.38/3.18
	ITR_CHR05_7491139	5	2019, 2020	4.48×10^{-13} /1.71×10^{-12}	***/***	3.84/3.56
	ITR_CHR05_7491762	5	2019, 2020	7.78×10^{-09} /2.43×10^{-09}	***/***	3.75/3.49
	ITR_CHR05_7491811	5	2019, 2020	7.49×10^{-10} /5.09×10^{-10}	***/***	4.36/4.01
	ITR_CHR05_7544355	5	2019, 2020	9.22×10^{-08} /7.64×10^{-08}	***/***	2.55/2.42
	ITR_CHR05_7602018	5	2019, 2020	5.79×10^{-07} /1.86×10^{-07}	***/***	3.75/3.64
	ITR_CHR05_7760991	5	2019, 2020	2.67×10^{-06} /5.23×10^{-06}	**/**	2.05/1.93
	ITR_CHR05_7761458	5	2019, 2020	1.94×10^{-14} /8.68×10^{-14}	***/***	4.38/4.01
	ITR_CHR05_7763191	5	2019, 2020	3.94×10^{-09} /3.54×10^{-09}	***/***	3.40/3.07
	ITR_CHR05_7763323	5	2019, 2020	3.68×10^{-08} /3.68×10^{-08}	***/***	2.93/2.64
	ITR_CHR05_7763363	5	2019, 2020	3.12×10^{-08} /3.74×10^{-08}	***/***	3.27/2.95
	ITR_CHR05_7919185	5	2019, 2020	1.08×10^{-06} /2.81×10^{-06}	***/***	3.50/3.07
	ITR_CHR05_7919269	5	2019, 2020	8.22×10^{-06} /9.62×10^{-06}	*/*	1.66/1.42
	ITR_CHR05_8082904	5	2019, 2020	5.54×10^{-13} /2.82×10^{-12}	***/***	4.33/3.92
	ITR_CHR05_8260520	5	2019, 2020	4.93×10^{-09} /1.37×10^{-08}	***/***	4.08/3.68
	ITR_CHR05_8260654	5	2019, 2020	3.82×10^{-09} /1.05×10^{-08}	***/***	4.19/3.79
	ITR_CHR05_9382060	5	2019, 2020	8.29×10^{-06} /1.83×10^{-06}	**/**	3.06/3.05
	ITR_CHR05_9382078	5	2020	4.99×10^{-06}	**	3.00
	ITR_CHR05_11878439	5	2019, 2020	1.39×10^{-05} /6.31×10^{-06}	***/***	3.48/3.28
	ITR_CHR05_11878459	5	2019, 2020	1.34×10^{-06} /3.68×10^{-07}	***/***	3.15/3.06
	ITR_CHR05_11878498	5	2019, 2020	2.94×10^{-07} /5.37×10^{-08}	***/***	3.39/3.20

Supplementary Table 1. Summary of the responsive SNP markers for the relative anthocyanin (AN) content above the *qqman* significance threshold (continue).

Population	Marker[a]	HG[b]	Year[c]	*p*-value (2019/2020)	ANOVA[d] (2019/2020)	MD[e] (2019/2020)
WSF_1	ITR_CHR05_11878509	5	2019, 2020	3.13×10^{-07} /6.84×10^{-08}	***/***	3.46/3.27
	ITR_CHR05_12844143	5	2019, 2020	1.21×10^{-09} /1.15×10^{-08}	***/***	4.17/3.77
	ITR_CHR05_12844145	5	2019, 2020	1.56×10^{-09} /1.34×10^{-08}	***/***	4.27/3.86
	ITR_CHR05_13667656	5	2019, 2020	8.51×10^{-07} /4.70×10^{-07}	***/***	3.55/3.36
	ITR_CHR05_13667835	5	2019, 2020	1.04×10^{-06} /6.21×10^{-07}	***/***	3.45/3.28
	ITR_CHR05_13667846	5	2019, 2020	1.14×10^{-06} /6.82×10^{-07}	***/***	3.40/3.23
	ITR_CHR05_13667876	5	2019, 2020	5.30×10^{-07} /3.92×10^{-07}	***/***	3.56/3.34
	ITR_CHR05_20739390	5	2019, 2020	4.38×10^{-06} /4.64×10^{-06}	***/***	3.21/3.04
	ITR_CHR05_21520645	5	2019, 2020	2.51×10^{-06} /6.94×10^{-07}	***/***	2.53/3.33
	ITR_CHR05_21825731	5	2019, 2020	6.82×10^{-06} /3.84×10^{-06}	***/***	3.46/3.40
	ITR_CHR05_21825755	5	2019, 2020	1.28×10^{-05} /4.22×10^{-05}	***/***	3.89/3.63
	ITR_CHR05_21825762	5	2019, 2020	6.58×10^{-06} /5.42×10^{-06}	***/***	2.87/2.62
	ITR_CHR05_21825771	5	2019, 2020	8.15×10^{-06} /1.79×10^{-06}	***/***	3.37/3.24
	ITR_CHR05_22049636	5	2019, 2020	6.21×10^{-06} /1.19×10^{-05}	***/***	3.48/3.24
	ITR_CHR05_22376550	5	2019, 2020	9.17×10^{-06} /8.13×10^{-06}	***/***	3.59/3.37
	ITR_CHR05_22405347	5	2019, 2020	4.88×10^{-07} /1.62×10^{-07}	***/***	3.84/3.62
	ITR_CHR05_22832191	5	2019, 2020	5.41×10^{-19} /1.04×10^{-18}	***/***	5.03/4.58
	ITR_CHR05_22897808	5	2019, 2020	8.95×10^{-07} /7.18×10^{-07}	*/*	2.36/1.97
	ITR_CHR05_23460474	5	2019, 2020	4.85×10^{-18} /4.35×10^{-18}	***/***	5.18/4.79
	ITR_CHR05_23460534	5	2019, 2020	1.07×10^{-07} /1.77×10^{-07}	**/*	2.66/2.18
	ITR_CHR05_23484740	5	2019, 2020	2.90×10^{-15} /1.86×10^{-15}	***/***	4.34/4.04

Supplementary Table 1. Summary of the responsive SNP markers for the relative anthocyanin (AN) content above the *qqman* significance threshold (continue).

Population	Marker[a]	HG[b]	Year[c]	*p*-value (2019/2020)	ANOVA[d] (2019/2020)	MD[e] (2019/2020)
WSF_1	ITR_CHR05_23484743	5	2019, 2020	8.03×10^{-06} /4.87×10^{-06}	***/***	2.88/2.73
	ITR_CHR05_23536069	5	2019, 2020	1.48×10^{-18} /2.98×10^{-18}	***/***	5.11/4.75
	ITR_CHR05_23551173	5	2019, 2020	1.09×10^{-19} /1.96×10^{-19}	***/***	4.84/4.42
	ITR_CHR05_23576424	5	2019, 2020	5.20×10^{-07} /5.78×10^{-06}	***/***	2.88/2.53
	ITR_CHR05_23576531	5	2019	3.95×10^{-06}	***	2.85
	ITR_CHR05_23834235	5	2019, 2020	1.32×10^{-13} /1.16×10^{-13}	***/***	4.15/3.89
	ITR_CHR05_24049567	5	2019, 2020	2.96×10^{-07} /3.14×10^{-07}	***/***	3.63/3.45
	ITR_CHR05_24049978	5	2019, 2020	1.49×10^{-07} /1.50×10^{-07}	***/***	3.88/3.71
	ITR_CHR05_24291939	5	2019, 2020	3.78×10^{-15} /2.67×10^{-15}	***/***	4.56/4.28
	ITR_CHR05_24291958	5	2019, 2020	7.01×10^{-15} /4.20×10^{-15}	***/***	4.63/4.34
	ITR_CHR05_24605925	5	2019, 2020	3.07×10^{-18} /2.05×10^{-18}	***/***	4.51/4.24
	ITR_CHR05_24605927	5	2019, 2020	3.34×10^{-08} /4.19×10^{-08}	***/***	4.32/4.02
	ITR_CHR05_24655796	5	2019, 2020	3.08×10^{-14} /1.23×10^{-13}	**/*	2.58/1.94
	ITR_CHR05_24655817	5	2019, 2020	3.11×10^{-07} /4.02×10^{-07}	*/*	2.40/1.96
	ITR_CHR05_24655895	5	2019, 2020	2.55×10^{-06} /3.51×10^{-06}	**/**	2.10/2.06
	ITR_CHR05_24667124	5	2019, 2020	7.64×10^{-06} /8.64×10^{-06}	***/***	3.43/3.21
	ITR_CHR05_24750065	5	2019, 2020	2.36×10^{-17} /1.11×10^{-16}	***/***	4.75/4.32
	ITR_CHR05_24753965	5	2019, 2020	1.22×10^{-08} /1.56×10^{-09}	***/***	4.86/4.57
	ITR_CHR05_24861975	5	2019, 2020	9.94×10^{-15} /3.43×10^{-14}	***/***	4.60/4.31
	ITR_CHR05_24862401	5	2019, 2020	2.91×10^{-20} /1.32×10^{-19}	***/***	5.82/5.43
	ITR_CHR05_24927289	5	2019, 2020	1.13×10^{-18} /1.85×10^{-18}	***/***	5.89/5.41

Supplementary Table 1. Summary of the responsive SNP markers for the relative anthocyanin (AN) content above the *qqman* significance threshold (continue).

Population	Marker[a]	HG[b]	Year[c]	*p*-value (2019/2020)	ANOVA[d] (2019/2020)	MD[e] (2019/2020)
WSF_1	ITR_CHR05_24927560	5	2019, 2020	1.83×10^{-18} /3.85×10^{-18}	***/***	5.81/5.30
	ITR_CHR05_24931541	5	2019, 2020	7.30×10^{-06} /1.26×10^{-05}	***/***	3.09/2.72
	ITR_CHR05_24960141	5	2019, 2020	9.62×10^{-20} /1.04×10^{-19}	***/***	5.39/4.98
	ITR_CHR05_24960765	5	2019, 2020	8.21×10^{-09} /9.04×10^{-09}	***/***	3.33/3.06
	ITR_CHR05_25000690	5	2019, 2020	1.18×10^{-11} /3.36×10^{-11}	***/***	4.87/4.41
	ITR_CHR05_25000760	5	2019	1.03×10^{-05}	***	2.67
	ITR_CHR05_25145392	5	2019, 2020	4.31×10^{-09} /6.60×10^{-09}	***/***	2.51/2.21
	ITR_CHR05_25145537	5	2019, 2020	5.87×10^{-09} /1.04×10^{-08}	***/***	2.57/2.27
	ITR_CHR05_25375645	5	2019, 2020	4.93×10^{-18} /5.64×10^{-18}	***/***	5.54/5.09
	ITR_CHR05_25375649	5	2019, 2020	4.61×10^{-18} /5.53×10^{-18}	***/***	5.54/5.09
	ITR_CHR05_25378105	5	2019, 2020	1.31×10^{-06} /3.02×10^{-07}	***/***	2.98/2.85
	ITR_CHR05_25439406	5	2019, 2020	1.45×10^{-14} /2.44×10^{-15}	***/***	5.01/4.61
	ITR_CHR05_25439533	5	2019, 2020	7.68×10^{-14} /1.12×10^{-14}	***/***	4.85/4.50
	ITR_CHR05_25439600	5	2019, 2020	1.11×10^{-13} /1.62×10^{-13}	***/***	4.19/3.91
	ITR_CHR05_25439632	5	2019, 2020	2.19×10^{-16} /3.93×10^{-16}	***/***	4.78/4.50
	ITR_CHR05_25701591	5	2019, 2020	6.22×10^{-23} /3.96×10^{-22}	***/***	5.37/4.90
	ITR_CHR05_25731262	5	2019, 2020	6.29×10^{-17} /6.72×10^{-16}	***/***	4.29/3.83
	ITR_CHR05_25832811	5	2019, 2020	3.86×10^{-14} /1.69×10^{-13}	***/***	5.25/4.75
	ITR_CHR05_26029224	5	2019, 2020	1.43×10^{-24} /6.12×10^{-24}	***/***	5.72/5.20
	ITR_CHR05_26275794	5	2019, 2020	2.23×10^{-25} /3.58×10^{-24}	***/***	5.9/5.35
	ITR_CHR05_26275798	5	2019, 2020	1.26×10^{-25} /1.81×10^{-24}	***/***	6.06/5.46
	ITR_CHR05_26275799	5	2019, 2020	6.54×10^{-26} /1.20×10^{-24}	***/***	5.97/5.42

Supplementary Table 1. Summary of the responsive SNP markers for the relative anthocyanin (AN) content above the *qqman* significance threshold (continue).

Population	Marker[a]	HG[b]	Year[c]	*p*-value (2019/2020)	ANOVA[d] (2019/2020)	MD[e] (2019/2020)
WSF$_1$	ITR_CHR05_26275800	5	2019, 2020	7.26×10^{-26} /1.24×10^{-24}	***/***	6.19/5.61
	ITR_CHR05_26275802	5	2019, 2020	1.35×10^{-25} /1.98×10^{-24}	***/***	6.05/5.48
	ITR_CHR05_26351002	5	2019, 2020	4.86×10^{-23} /2.80×10^{-23}	***/***	5.70/5.32
	ITR_CHR05_26376097	5	2019, 2020	6.48×10^{-09} /3.51×10^{-09}	***/***	3.33/3.22
	ITR_CHR05_26387002	5	2019, 2020	2.66×10^{-17} /4.48×10^{-18}	***/***	5.75/5.40
	ITR_CHR05_26396741	5	2019, 2020	1.18×10^{-28} /3.19×10^{-28}	***/***	6.08/5.59
	ITR_CHR05_26461536	5	2019, 2020	4.23×10^{-21} /1.22×10^{-20}	***/***	5.88/5.33
	ITR_CHR05_26461587	5	2019, 2020	2.16×10^{-09} /1.33×10^{-09}	***/***	3.55/3.24
	ITR_CHR05_26470283	5	2019, 2020	5.81×10^{-10} /6.44×10^{-10}	***/***	5.33/4.90
	ITR_CHR05_26499551	5	2019, 2020	9.21×10^{-09} /8.83×10^{-09}	***/***	3.68/3.30
	ITR_CHR05_26504596	5	2019, 2020	7.30×10^{-13} /1.32×10^{-11}	***/***	4.24/3.77
	ITR_CHR05_26504674	5	2019, 2020	1.67×10^{-10} /1.34×10^{-10}	***/***	4.35/4.05
	ITR_CHR05_26504679	5	2019, 2020	4.22×10^{-20} /1.32×10^{-19}	***/***	6.18/5.75
	ITR_CHR05_26504686	5	2019, 2020	1.04×10^{-19} /3.77×10^{-19}	***/***	6.18/5.75
	ITR_CHR05_26504703	5	2019, 2020	1.38×10^{-20} /6.85×10^{-20}	***/***	6.20/5.74
	ITR_CHR05_26504711	5	2019, 2020	6.70×10^{-21} /2.45×10^{-20}	***/***	6.18/5.77
	ITR_CHR05_26504850	5	2019, 2020	1.68×10^{-06} /5.34×10^{-07}	***/**	2.60/2.58
	ITR_CHR05_26504916	5	2019, 2020	1.48×10^{-06} /5.47×10^{-07}	***/**	2.68/2.63
	ITR_CHR05_26549861	5	2019, 2020	5.79×10^{-07} /5.36×10^{-07}	***/***	3.35/3.21
	ITR_CHR05_26550111	5	2019, 2020	1.25×10^{-15} /5.97×10^{-16}	***/***	3.95/3.74
	ITR_CHR05_26550117	5	2019, 2020	1.03×10^{-15} /4.91×10^{-16}	***/***	4.00/3.78

Supplementary Table 1. Summary of the responsive SNP markers for the relative anthocyanin (AN) content above the *qqman* significance threshold (continue).

Population	Marker[a]	HG[b]	Year[c]	*p*-value (2019/2020)	ANOVA[d] (2019/2020)	MD[e] (2019/2020)
WSF_1	ITR_CHR05_26550141	5	2019, 2020	2.15×10^{-12} /5.24×10^{-13}	***/***	3.89/3.64
	ITR_CHR05_26550193	5	2019, 2020	3.25×10^{-15} /2.72×10^{-14}	***/***	5.24/4.62
	ITR_CHR05_26563505	5	2019, 2020	8.83×10^{-17} /1.70×10^{-16}	***/***	5.44/4.95
	ITR_CHR05_26631595	5	2019, 2020	3.59×10^{-07} /3.65×10^{-06}	***/***	3.28/2.81
	ITR_CHR05_26701812	5	2019, 2020	1.64×10^{-25} /4.31×10^{-25}	***/***	5.65/5.16
	ITR_CHR05_26701979	5	2019, 2020	1.73×10^{-25} /4.79×10^{-25}	***/***	5.85/5.35
	ITR_CHR05_26718188	5	2019, 2020	4.48×10^{-27} /1.65×10^{-26}	***/***	6.29/5.82
	ITR_CHR05_26718670	5	2019, 2020	8.39×10^{-26} /7.33×10^{-26}	***/***	6.08/5.61
	ITR_CHR05_26863677	5	2019, 2020	6.13×10^{-07} /3.06×10^{-06}	***/***	3.54/3.08
	ITR_CHR05_26863693	5	2019, 2020	9.80×10^{-07} /5.72×10^{-06}	***/***	3.74/3.22
	ITR_CHR05_26863734	5	2019	3.28×10^{-06}	***	3.64
	ITR_CHR05_26864099	5	2019, 2020	1.86×10^{-19} /4.41×10^{-19}	***/***	6.55/5.86
	ITR_CHR05_26995149	5	2019, 2020	2.67×10^{-06} /1.01×10^{-06}	**/**	3.83/3.57
	ITR_CHR05_27046380	5	2019, 2020	1.71×10^{-27} /2.97×10^{-27}	***/***	7.06/6.49
	ITR_CHR05_27046381	5	2019, 2020	4.18×10^{-28} /7.74×10^{-28}	***/***	7.22/6.64
	ITR_CHR05_27046400	5	2019, 2020	9.11×10^{-28} /1.53×10^{-27}	***/***	6.77/6.21
	ITR_CHR05_27046418	5	2019, 2020	1.17×10^{-27} /2.01×10^{-27}	***/***	7.15/6.57
	ITR_CHR05_27090704	5	2019, 2020	1.05×10^{-30} /1.05×10^{-30}	***/***	6.24/5.81
	ITR_CHR05_27090748	5	2019, 2020	5.75×10^{-10} /2.34×10^{-10}	***/***	3.97/3.70
	ITR_CHR05_27090781	5	2019, 2020	8.15×10^{-10} /3.14×10^{-10}	***/***	4.01/3.73
	ITR_CHR05_27149467	5	2019, 2020	6.90×10^{-06} /8.14×10^{-06}	***/***	3.96/3.68
	ITR_CHR05_27203861	5	2019, 2020	1.51×10^{-06} /5.88×10^{-07}	***/***	3.04/2.77

Supplementary Table 1. Summary of the responsive SNP markers for the relative anthocyanin (AN) content above the *qqman* significance threshold (continue).

Population	Marker[a]	HG[b]	Year[c]	*p*-value (2019/2020)	ANOVA[d] (2019/2020)	MD[e] (2019/2020)
WSF_1	ITR_CHR05_27314316	5	2019, 2020	$5.57×10^{-10}$ /$2.26×10^{-09}$	***/***	3.83/3.55
	ITR_CHR05_27314332	5	2019, 2020	$8.55×10^{-10}$ /$3.85×10^{-09}$	***/***	3.69/3.43
	ITR_CHR05_27314520	5	2019, 2020	$2.76×10^{-12}$ /$5.52×10^{-13}$	***/***	4.25/3.97
	ITR_CHR05_27324332	5	2019, 2020	$8.62×10^{-07}$ /$3.63×10^{-06}$	***/***	3.56/3.10
	ITR_CHR05_27388420	5	2019, 2020	$4.57×10^{-20}$ /$2.96×10^{-20}$	***/***	5.54/5.24
	ITR_CHR05_27388441	5	2019, 2020	$1.54×10^{-19}$ /$1.66×10^{-19}$	***/***	5.48/5.16
	ITR_CHR05_27388444	5	2019, 2020	$7.63×10^{-20}$ /$4.37×10^{-20}$	***/***	5.72/5.38
	ITR_CHR05_27388642	5	2019, 2020	$1.11×10^{-19}$ /$7.47×10^{-20}$	***/***	5.64/5.32
	ITR_CHR05_27423535	5	2019, 2020	$4.36×10^{-09}$ /$6.87×10^{-09}$	***/***	4.03/3.70
	ITR_CHR05_27423536	5	2019, 2020	$3.03×10^{-09}$ /$5.68×10^{-09}$	***/***	4.07/3.74
	ITR_CHR05_27423555	5	2019, 2020	$5.60×10^{-10}$ /$1.31×10^{-09}$	***/***	3.96/3.64
	ITR_CHR05_27424024	5	2019, 2020	$5.19×10^{-09}$ /$7.04×10^{-09}$	***/***	3.65/3.32
	ITR_CHR05_27424035	5	2019, 2020	$2.31×10^{-09}$ /$3.32×10^{-09}$	***/***	4.19/3.85
	ITR_CHR05_27427060	5	2019, 2020	$5.89×10^{-10}$ /$2.29×10^{-10}$	***/***	3.97/3.75
	ITR_CHR05_27569615	5	2019, 2020	$3.41×10^{-24}$ /$1.39×10^{-23}$	***/***	5.23/4.78
	ITR_CHR05_27666689	5	2019, 2020	$2.34×10^{-09}$ /$1.27×10^{-09}$	***/***	3.85/3.51
	ITR_CHR05_27696681	5	2019, 2020	$5.53×10^{-18}$ /$2.04×10^{-17}$	***/***	5.31/4.93
	ITR_CHR05_27835691	5	2019, 2020	$6.54×10^{-09}$ /$3.92×10^{-09}$	***/***	3.41/3.27
	ITR_CHR05_27879351	5	2019, 2020	$2.11×10^{-08}$ /$3.07×10^{-08}$	***/***	3.75/3.53
	ITR_CHR05_27879726	5	2019, 2020	$4.85×10^{-22}$ /$4.72×10^{-22}$	***/***	5.85/5.38
	ITR_CHR05_28113058	5	2019, 2020	$7.10×10^{-09}$ /$1.17×10^{-09}$	***/***	3.43/3.30

Supplementary Table 1. Summary of the responsive SNP markers for the relative anthocyanin (AN) content above the *qqman* significance threshold (continue).

Population	Marker[a]	HG[b]	Year[c]	*p*-value (2019/2020)	ANOVA[d] (2019/2020)	MD[e] (2019/2020)
WSF_1	ITR_CHR05_28113478	5	2019, 2020	1.10×10^{-06} /7.51×10^{-08}	***/***	3.46/3.28
	ITR_CHR05_28113490	5	2019, 2020	6.10×10^{-07} /5.52×10^{-08}	***/***	3.22/3.06
	ITR_CHR05_28124449	5	2019, 2020	7.79×10^{-09} /8.27×10^{-10}	***/***	3.37/3.10
	ITR_CHR05_28124560	5	2019, 2020	6.09×10^{-09} /1.12×10^{-09}	***/***	3.42/3.15
	ITR_CHR05_28124575	5	2019, 2020	5.03×10^{-06} /6.38×10^{-06}	*/*	3.17/2.82
	ITR_CHR05_28124576	5	2019, 2020	6.19×10^{-06} /6.77×10^{-06}	***/***	3.63/3.30
	ITR_CHR05_28124683	5	2019, 2020	1.79×10^{-09} /1.06×10^{-09}	***/***	3.11/2.98
	ITR_CHR05_28447653	5	2019, 2020	2.06×10^{-08} /1.33×10^{-08}	***/***	2.98/2.89
	ITR_CHR05_28508098	5	2019, 2020	1.38×10^{-06} /9.87×10^{-07}	***/***	2.82/2.68
	ITR_CHR05_28530402	5	2019, 2020	6.54×10^{-06} /1.09×10^{-07}	***/***	2.95/2.88
	ITR_CHR05_28622236	5	2019, 2020	1.51×10^{-06} /1.60×10^{-06}	***/***	2.65/2.58
	ITR_CHR05_28700370	5	2019, 2020	2.50×10^{-07} /9.90×10^{-07}	***/***	2.91/2.68
	ITR_CHR05_28700379	5	2019, 2020	2.57×10^{-07} /1.02×10^{-06}	***/***	2.91/2.68
	ITR_CHR05_28700444	5	2019, 2020	4.20×10^{-07} /1.32×10^{-06}	***/***	2.94/2.78
	ITR_CHR05_28700767	5	2019, 2020	3.23×10^{-07} /1.32×10^{-06}	***/***	2.94/2.77
	ITR_CHR05_28700784	5	2019, 2020	7.87×10^{-07} /3.10×10^{-06}	***/***	2.79/2.56
	ITR_CHR05_28745289	5	2019, 2020	2.07×10^{-13} /2.15×10^{-13}	***/***	4.15/3.80
	ITR_CHR05_28980962	5	2019, 2020	6.12×10^{-06} /1.22×10^{-06}	***/***	3.05/2.97
	ITR_CHR05_28980993	5	2019, 2020	7.06×10^{-06} /1.52×10^{-06}	***/***	3.00/2.92
	ITR_CHR05_29049932	5	2019, 2020	1.08×10^{-12} /2.17×10^{-12}	***/***	3.75/4.08
	ITR_CHR05_29195061	5	2019, 2020	2.59×10^{-6} /2.04×10^{-7}	***/***	3.02/2.81

Supplementary Table 1. Summary of the responsive SNP markers for the relative anthocyanin (AN) content above the *qqman* significance threshold (continue).

Population	Marker[a]	HG[b]	Year[c]	*p*-value (2019/2020)	ANOVA[d] (2019/2020)	MD[e] (2019/2020)
WSF_1	ITR_CHR05_29257740	5	2019, 2020	6.01×10^{-07} /5.03×10^{-07}	***/***	2.88/2.73
	ITR_CHR05_29358196	5	2019, 2020	2.18×10^{-12} /4.70×10^{-12}	***/***	4.16/3.83
	ITR_CHR05_29385059	5	2019, 2020	7.37×10^{-13} /1.53×10^{-12}	***/***	3.93/3.61
	ITR_CHR05_29394223	5	2019, 2020	9.79×10^{-14} /3.66×10^{-13}	***/***	3.93/3.59
	ITR_CHR05_29451144	5	2019, 2020	2.13×10^{-12} /4.26×10^{-12}	***/***	3.65/3.30
	ITR_CHR05_29831079	5	2019, 2020	1.34×10^{-06} /4.82×10^{-06}	***/***	3.80/4.46
	ITR_CHR06_20062844	6	2019, 2020	1.79×10^{-06}/ 1.21×10^{-06}	***/***	3.94/3.64
	ITR_CHR07_17084932	7	2019, 2020	6.94×10^{-21} /1.87×10^{-20}	***/***	6.07/5.42
	ITR_CHR07_17084999	7	2019, 2020	9.43×10^{-21} /2.25×10^{-20}	***/***	6.17/5.52
	ITR_CHR07_17085050	7	2019, 2020	4.24×10^{-20} /1.06×10^{-19}	***/***	5.98/5.35
	ITR_CHR07_17085088	7	2019, 2020	9.69×10^{-19} /2.10×10^{-18}	***/***	5.38/4.80
	ITR_CHR07_17085099	7	2019, 2020	1.22×10^{-20} /2.86×10^{-20}	***/***	6.02/5.38
	ITR_CHR07_17085109	7	2019, 2020	1.21×10^{-20} /2.86×10^{-20}	***/***	6.08/5.43
	ITR_CHR07_17548278	7	2019, 2020	8.46×10^{-20} /6.38×10^{-20}	***/***	6.16/5.67
	ITR_CHR07_17548345	7	2019, 2020	9.24×10^{-20} /6.22×10^{-20}	***/***	6.27/5.76
	ITR_CHR07_17548396	7	2019, 2020	1.18×10^{-19} /8.80×10^{-20}	***/***	6.16/5.66
	ITR_CHR07_17548434	7	2019, 2020	5.10×10^{-18} /4.90×10^{-18}	***/***	5.83/5.29
	ITR_CHR07_17548445	7	2019, 2020	2.10×10^{-20} /1.54×10^{-20}	***/***	6.32/5.79
	ITR_CHR07_17548455	7	2019, 2020	3.47×10^{-20} /2.31×10^{-20}	***/***	6.23/5.73
	ITR_CHR10_24205086	10	2019	7.99×10^{-06}	**	2.16
	ITR_CHR13_13144677	13	2019, 2020	1.72×10^{-17} /2.80×10^{-17}	***/***	4.53/4.12

Supplementary Table 1. Summary of the responsive SNP markers for the relative anthocyanin (AN) content above the *qqman* significance threshold (continue).

Population	Marker[a]	HG[b]	Year[c]	*p*-value (2019/2020)	ANOVA[d] (2019/2020)	MD[e] (2019/2020)
WSF_1	ITR_CHR13_13144678	13	2019, 2020	5.76×10^{-17} / 7.89×10^{-17}	***/***	4.46/4.05
	ITR_CHR13_13144689	13	2019, 2020	6.65×10^{-07} / 1.97×10^{-06}	**/*	2.54/1.90
	ITR_CHR13_13144896	13	2019, 2020	5.07×10^{-17} / 6.44×10^{-17}	***/***	4.00/3.58
	ITR_CHR15_8725114	15	2019, 2020	2.57×10^{-13} / 8.83×10^{-14}	***/***	4.40/4.07
	ITR_CHR15_8725486	15	2019, 2020	8.08×10^{-13} / 3.37×10^{-13}	***/***	4.23/3.94
HAF_1	ITR_CHR09_1235154	9	2019	6.99×10^{-06}	***	5.28
LAF_1	NA					

[a]Marker order was sorted by position on the HG. 'ITR' indicates, *Ipomoea trifida*, 'CHR' indicates the chromosome and 'numeric' indicates the position (bp) of each marker on its respective chromosome.
[b]HG indicates homologous group.
[c]Year 2018 is data of relative AN from 94 KAF_1 population grown under pot condition (Haque et al., 2020b)
[d]*, ** and *** indicate significant differences between genotypes by ANOVA at $P < 0.05$, $P < 0.01$ and $P < 0.001$
[e]MD indicates the mean difference of the relative AN content of the F_1 population according to the homozygous and heterozygous genotypes of the SNPs markers, respectively.

Supplementary Table 2. Homologous position of starch metabolizing genes in sweetpotato genome.

Name[a]	Gene ID/ Accession no.	HG[b] (*I. trifida*)	Position within *I. trifida*[c]
Enzyme genes			
Ipomoea batatas sucrose synthase (IbSUS)	EU908020.1	1	26731433-26734645
		3	31667299-31670695
		9	4522837-4525420
Ipomoea trifida sucrose synthase1-1 (ItSUS1-1)	Itr_sc000040.1_g00060.1	9	4511289-4522985
ItSUS1-2	Itr_sc000306.1_g00010.1	9	23756114-23767860
ItSUS2	Itr_sc037738.1_g00001.1	6	6557619-6569230
ItSUS3	Itr_sc000158.1_g00019.1	12	31880480-31886824
		9	4522911-4523381
		1	26733539-26734020
ItSUS4-1	Itr_sc000040.1_g00062.1	9	4510902-4526153
		3	31668142-31670695
		1	26731436-26734083
ItSUS4-2	Itr_sc000092.1_g00028.1	1	26730944-26734318
		3	31668143-31670695
		9	4522837-4525608
ItSUS4-3	Itr_sc001186.1_g00008.1	3	31667553-31670941
		1	26731433-26734083
		9	4522488-4525612
ItSUS6-1	Itr_sc000509.1_g00004.1	11	2293808-2297920
ItSUS6-2	Itr_sc000564.1_g00008.1	3	32788252-32791882
		2	2975391-2975884
		11	2297145-2297669
ItSUS6-3	Itr_sc000793.1_g00030.1	2	2975113-2978602
		11	2293830-2297702
		3	32788857-32789415
Ipomoea batatasUDP-glucose pyrophosphorylase (IbUGPase)	EU863220.1	5	1139643-1139938
Ipomoea trifida UDP-glucose pyrophosphorylase2-1 (ItUGPase2-1)	Itr_sc000005.1_g00038.1	5	1141175-1144818
ItUGPase2-2	Itr_sc003340.1_g00004.1	13	5186990-5187138
ItUGPase3	Itr_sc000654.1_g00003.1	2	29696723-29702274
Ipomoea batata beta-fructofuranosidase1 (Ibβfruct1)	AF017082	2	30403436-30404851
		11	8800503-8804904
		9	6885591-6885904
		8	6138515-6139373
		13	24793390-24794248
Ipomoea trifida beta-fructofuranosidase1-1 (ItFruct1-1)	Itr_sc000069.1_g00011.1	3	32163833-32167060
		1	26190862-26191683

Supplementary Table 2. Homologous position of starch metabolizing genes in sweetpotato genome (continue).

**Name**[a]	**Gene ID/ Accession no.**	**HG**[b] **(*I. trifida*)**	**Position within *I. trifida***[c]
	Enzyme genes		
ItFruct1-2	Itr_sc000069.1_g00022.1	3	32110028-32166328
		1	26190868-26191736
ItFruct1-3	Itr_sc000975.1_g00003.1	1	26190313-26192993
Ibβfruct2	AY037937	2	30401398-30405775
		11	8800503-8801364
		8	6138515-6138902
		13	24793390-24794248
Ibβfruct3	AY037938	11	8796767-8804883
		2	30403436-30404299
		8	6138523-6139378
		13	24793390-24794193
ItFruct4-1 (ref. Solanum tuberosum)	Itr_sc001880.1_g00001.1 (ref. XP_006355490.1)	13	24791394-24796697
		11	8800503-8801364
		2	30403436-30404300
		4	29192821-29193985
ItFruct4-2	Itr_sc002517.1_g00001.1	4	29191779-29195882
		13	24793390-24794248
		11	8800503-8801364
		2	30403436-30404300
Ipomoea batatas ADP-glucose pyrophosphorylase small subunit1 (IbAGPase small subunit1)	JQ797696	9	1716618-1720312
		3	27550494-27551659
IbAGPase small subunit2	JQ797697	3	27548966-27552745
		9	1718030-1719096
IbAGPase large subunit1	JQ797692	4	578153-580197
		6	579415-579587
		14	28931458-28931623
IbAGPase large subunit2	JQ797693	6	578579-580839
		4	578940-579113
		14	28931458-28931630
IbAGPase large subunit3	JQ797694	1	28904793-28907122
IbAGPase large subunit4	JQ797701	13	2078550-2081716
Ipomoea batatas starch phosphorylase (IbPHO)	M64362.1	11	6693824-6712325
Ipomoea batatas granule-bound starch synthase I (IbGBSSI)	**AB071604**	**15**	**5991269-5994843**
Ipomoea batatas starch synthase II (IbSSII)	AF068834	5	4202865-4206989
		2	730773-734082
Solanum tuberosum starch synthase III (StSSIII)	X94400.1	14	2015693-2018641

Supplementary Table 2. Homologous position of starch metabolizing genes in sweetpotato genome (continue).

**Name**[a]	**Gene ID/ Accession no.**	**HG**[b] **(*I. trifida*)**	**Position within *I. trifida***[c]
	Enzyme genes		
Ipomoea trifida SSIV-1 (ItSSIV-1)	Itr_sc000219.1_g00034.1	11	267932-269073
ItSSIV-2 (ref. S. tuberosum)	Itr_sc000329.1_g00007.1(ref. XP_006353746.1)	14	32914803-32915966
ItSSIV-3 (ref. S. tuberosum)	Itr_sc000329.1_g00008.1(ref. XP_006353746.1)	14	32903881-32908634
ItSSIV-4	Itr_sc001370.1_g00016.1	6	29327175-29335238
Ipomoea batatas starch branching enzymeI-1A (IbSBEI-1A)	AB194722	8	193389-199631
IbSBEI-1B	AB194723	8	193368-199631
IbSBEI-1C	AB194724	8	193354-199631
IbSBEI-2A	AB194725	8	193389-199498
IbSBEI-2B	AB194726	8	193389-199498
IbSBEII	AB071286	10	631028-641198
		8	195724-197026
Oryza sativa SBEII (OsSBEIII)	D16201	8	196278- 197029
Ipomoea batatas isoamylaseI (IbIsaI)	LC052789	2	23324044-23344647
IbIsaII	LC052790	12	11787412-11790232
IbIsaIII	LC052791	14	35771304-35781855
Solanum tuberosum pullulanase 1, chloroplastic (StPUL1)	XM_006361645.2	-	No homology region was found
Ipomoea batatas α-amylase / Ipomoea trifida α-amylase 1A (Ibα-amylase /ItAMY1A)	KT281131.1/ Itr_sc000017.1_g00087.1	4	2818222-2830900
		15	3190661-3195428
ItAMY1B	Itr_sc000017.1_g00088.1	4	2818222-2830492
		15	3190125-3195428
ItAMY1C	Itr_sc000017.1_g00090.1	4	2818236-2830900
		15	3190139-3195423
ItAMY1D	**Itr_sc000553.1_g00024.1**	**15**	**3190125-3192261**
		4	2822108-2830492
ItAMY1E	**Itr_sc000553.1_g00023.1**	**15**	**3193662-3195458**
		1	7145004-7145437
		4	2818651-2823114
ItAMY2A	Itr_sc001021.1_g00004.1	10	6493053-6497096
ItAMY2B (ref. S. tuberosum)	Itr_sc003247.1_g00001.1 (ref. XP_006363428.1)	11	6626194-6633404
ItAMY3	**Itr_sc000559.1_g00005.1**	10	8932228-8935369
		**15**	**20494944-20501917**
Ipomoea batatas Sporamin	U12436	14	999636-1097668

Supplementary Table 2. Homologous position of starch metabolizing genes in sweetpotato genome (continue).

**Name[a]**	**Gene ID/ Accession no.**	**HG[b] (*I. trifida*)**	**Position within *I. trifida*[c]**
	Transcription factors		
Ipomoea batatas Dof zinc finger protein1 (IbSRF1)	AB469355	3	21280012-21282954
		9	9631031-9632207
		2	746186-747291
Solanum tuberosum sucrose nonfermenting1-related protein kinase1 (StSnRK1)	KR069088.1	11	6820912- 6823904
Ipomoea batatas MADS-box protein1 (IbMADS1)	AF396746	2	5633872- 5638641
Ipomoea batatas nucleus accumbens-associated protein 1 (IbNAC1)	GQ280387	5	30190431-30191601
	Transporter genes		
Solanum tuberosum ATP/ADP-transporter (StAATP)	**Y10821**	**15**	**318247-321635**
		9	6034762-6037927

[a]Homology searches against ITR r2.2 were performed with BLAST program using the above sequences as queries.
[b]HG indicates, homologous group. Genes located on HG15 were shown in bold and listed in the Table 4. With respect to *ItAMY1A*, *ItAMY1B* and *ItAMY1C,* although the second hit was found in HG 15, all the possible primer sets made from these homologous regions were matched in HG4 and hence were not included in the candidate gene list of HG 15.
[c]Homologous position covering all the matched regions with high sequence identity in ITR r2.2 genome.

Supplementary Table 3. Primer pairs used in the real time PCR.

Gene	Primer	Sequences (5′-3′)	Product size (bp)
IbGBSSI	Forward	ATGACAAGCCAGTGAAGGGG	208
	Reverse	TCAGTTGCAGGGTTCCACTC	
IbAmy1D	Forward	TTGAAGGATTCCGCCGGAAA	106
	Reverse	GCCAGTGGCTTTGTGTTGAT	
IbAmy1E	Forward	GTGGAAATCTTGGCAGCTGA	148
	Reverse	TCCCACACTGCAAAGTCTTTG	
IbAmy3	Forward	CATTCTTTCCGGCGAACACC	176
	Reverse	TCGATAACGGTGGAGCTAGC	
AATP	Forward	TTGGAGGAGAGCAGGAGTCA	158
	Reverse	TCCTTTTGCTGTGACCACCA	
IbActin	Forward	TGTTAGCAACTGGGATGATATGG	199
	Reverse	GGATAGCACAGCCTGAATAGC	

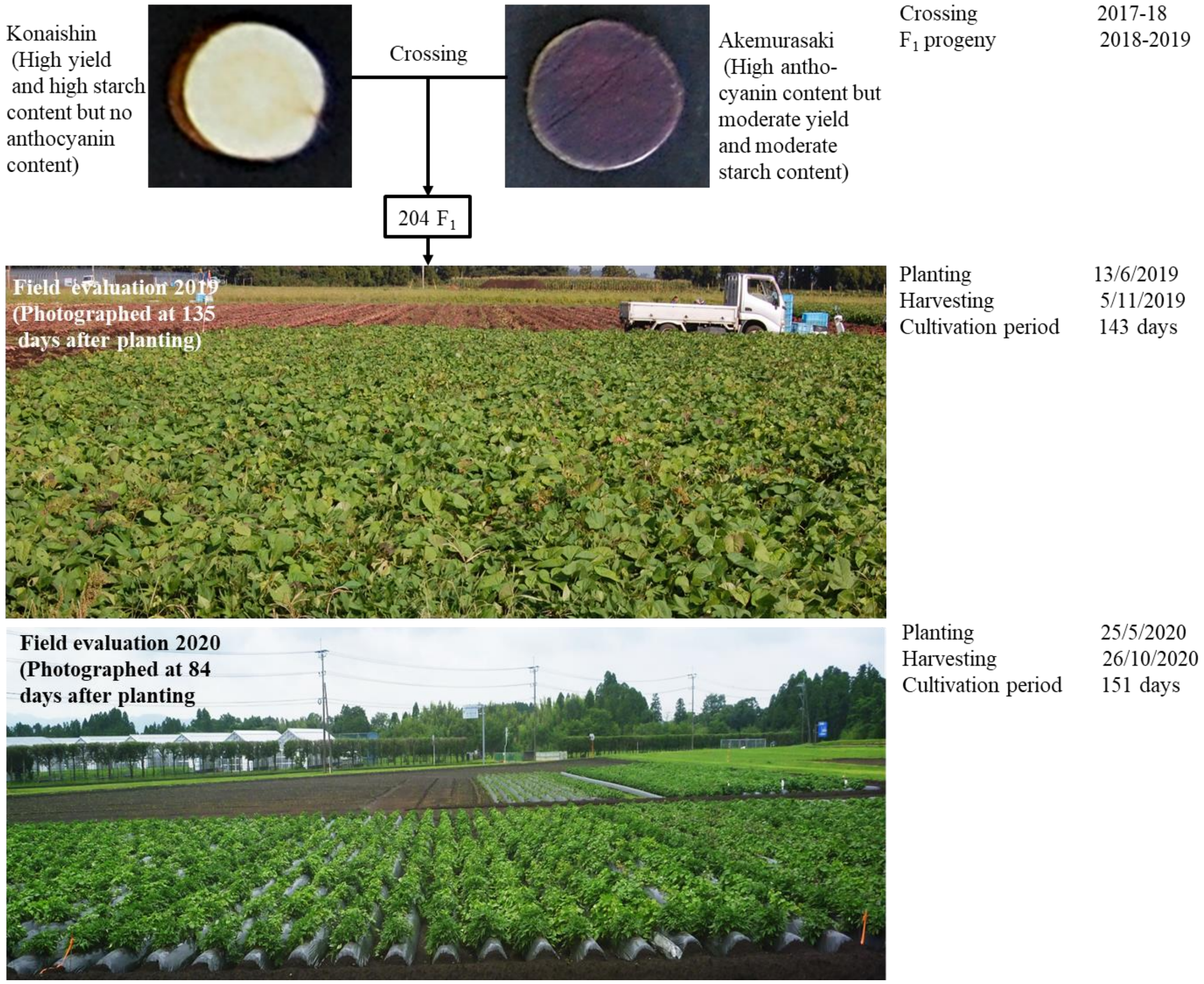

Supplementary Figure 1. Scheme of sweetpotato material development and field evaluation at Kyushu Okinawa Agricultural Research Center, NARO (KARC/NARO), Miyakonojo, Miyazaki prefecture, Japan.

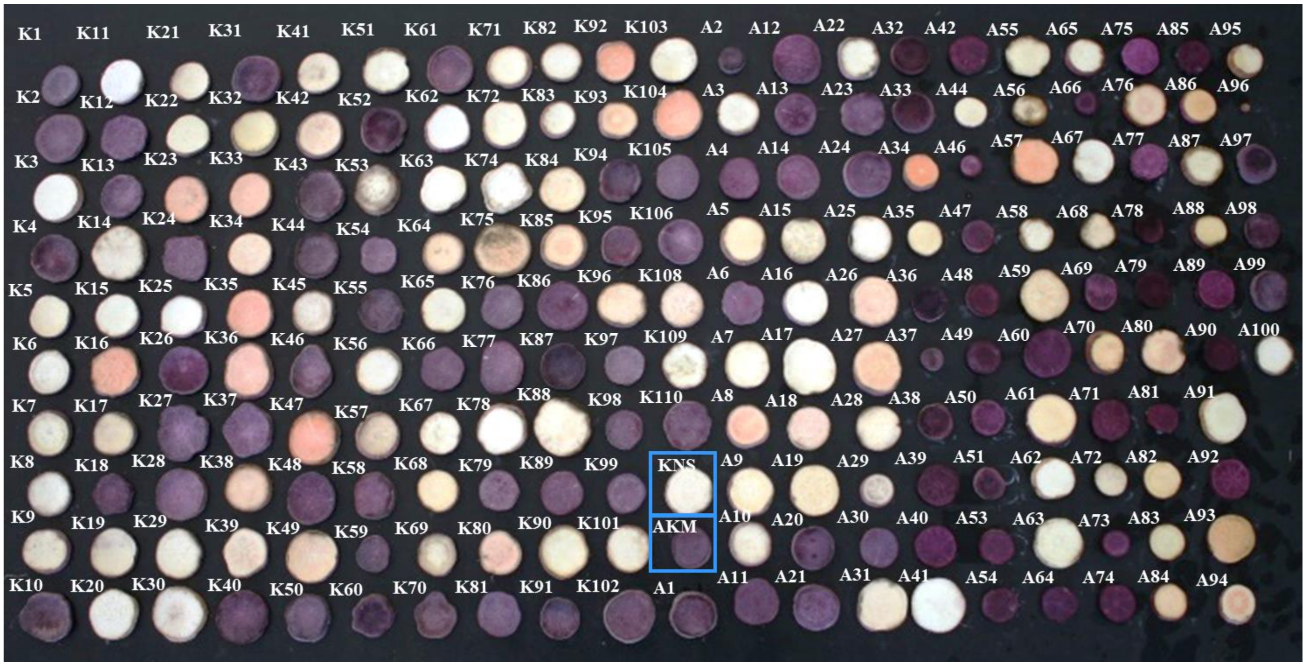

Supplementary Figure 2. Images of sliced storage roots in the Konaishin (KNS) × Akemurasaki (AKM) progenies. Photographs are from one replication as a representative of three replications.

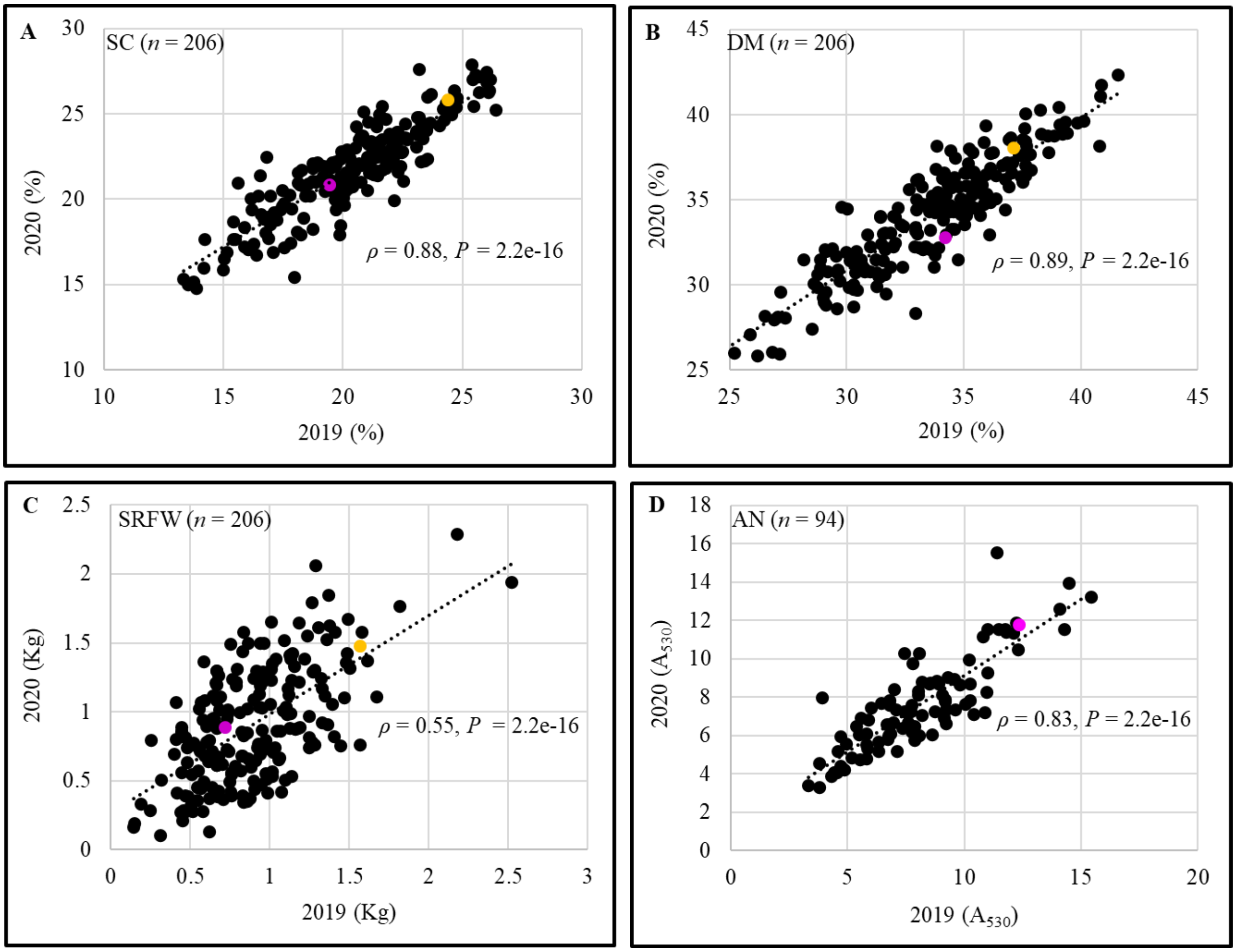

Supplementary Figure 3. X/Y plot comparison between 2019 and 2020 data of the F_1 population. **(A)** SC (*n* = 206). **(B)** DM (*n* = 206)). **(C)** SRFW (*n* = 206). **(D)** AN (*n* = 39). KNS and AKM were indicated with orange and purple color, respectively.

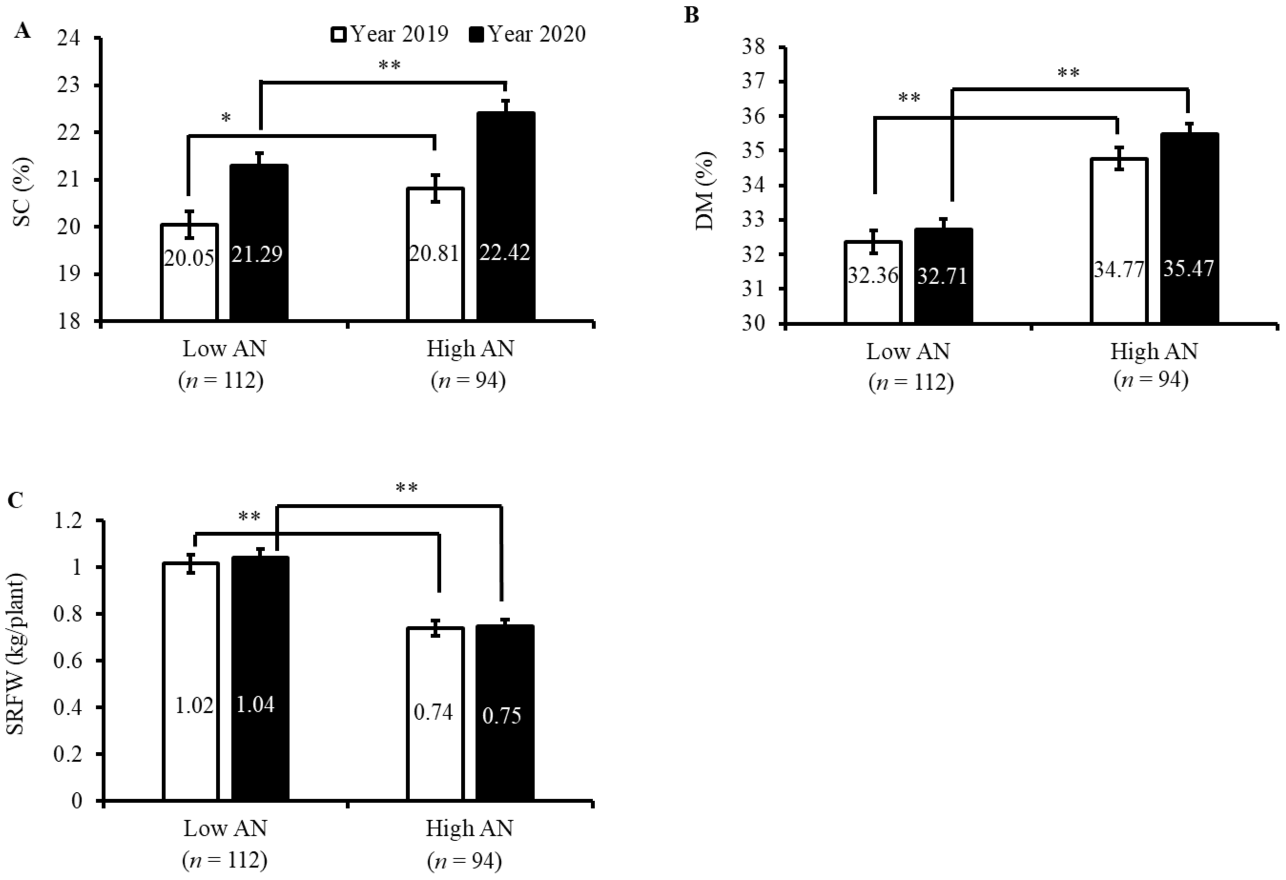

Supplementary Figure 4. Comparison on SC **(A)**, DM **(B)** and SRFW **(C)** of high AN containing 94 individuals (93 F_1 population + AKM) with low AN containing 112 individuals (111 F_1 population + KNS). Asterisk and double asterisk indicate significant differences at $p < 0.05$ and $p < 0.01$ based on the t test, respectively.

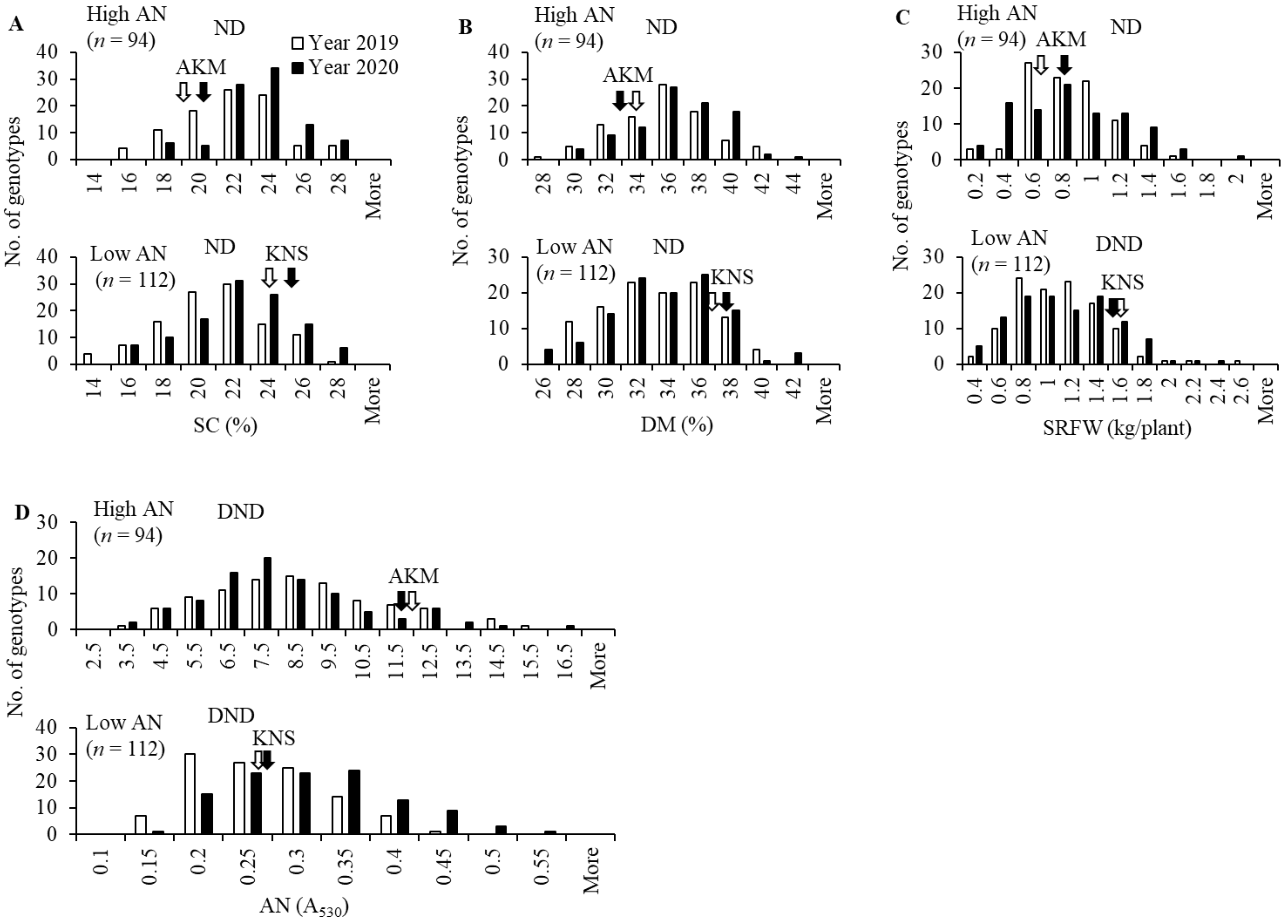

Supplementary Figure 5. Frequency distribution of breeding traits in the high AN containing 94 individuals (93 F_1 population + AKM) with low AN containing 112 individuals (111 F_1 population + KNS) in 2019 and 2020. **(A)** SC (%; *n* = 206). **(B)** DM (%; *n* = 206). **(C)** SRFW (kg/plant; *n* = 206). **(D)** AN (A_{530}; *n* = 206). Normality were cheeked with Shapiro-Wilk test at α = 0.05. ND, normal distribution; DND, distortion of normal distribution.

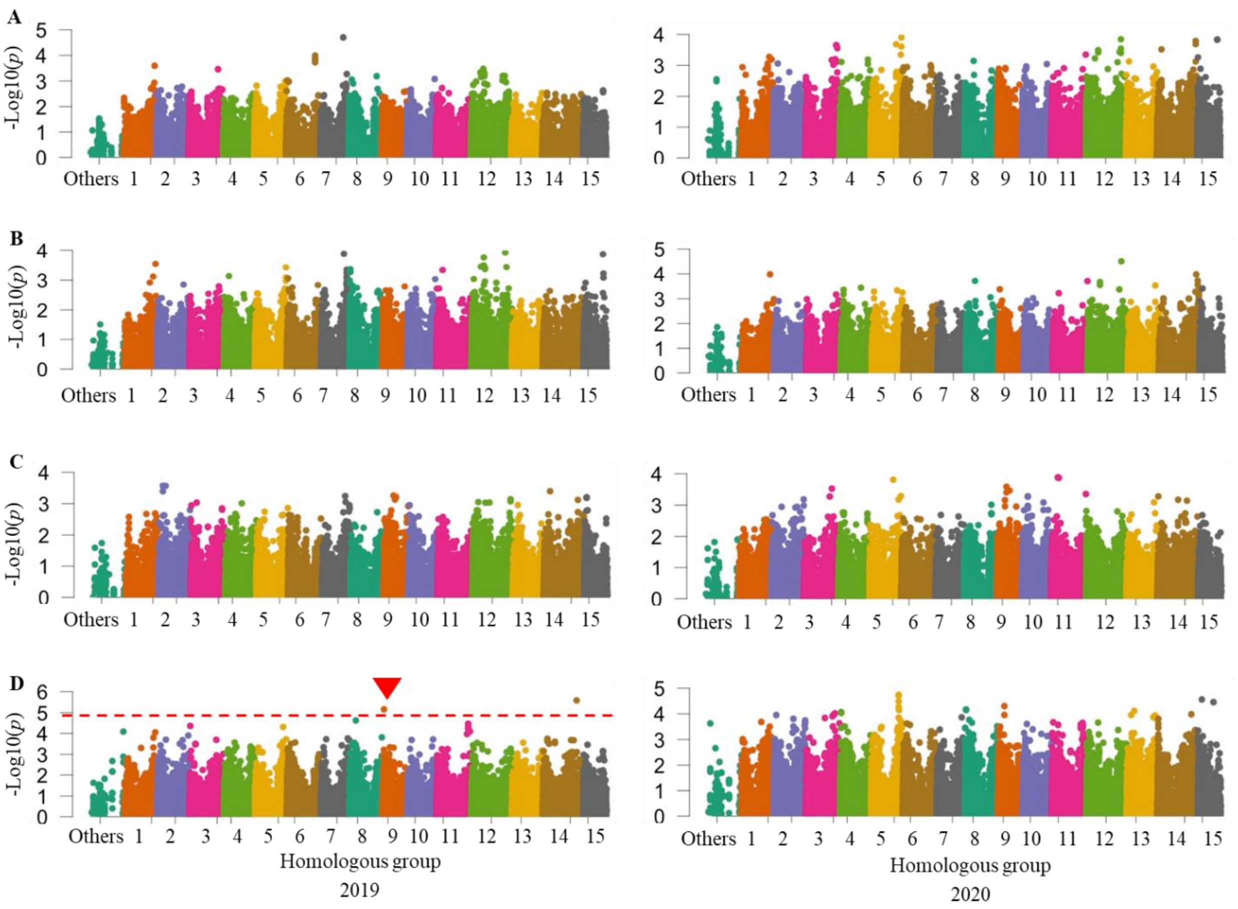

Supplementary Figure 6. Manhattan plots in the high AN containing 93 F_1 (HAF_1) population. **(A)** SC (%). **(B)** DM (%). **(C)** SRFW (kg/plant). **(D)** AN. The horizontal dashed red line represents the significance thresholds. The arrowhead indicates the location of the signal having significant SNP.

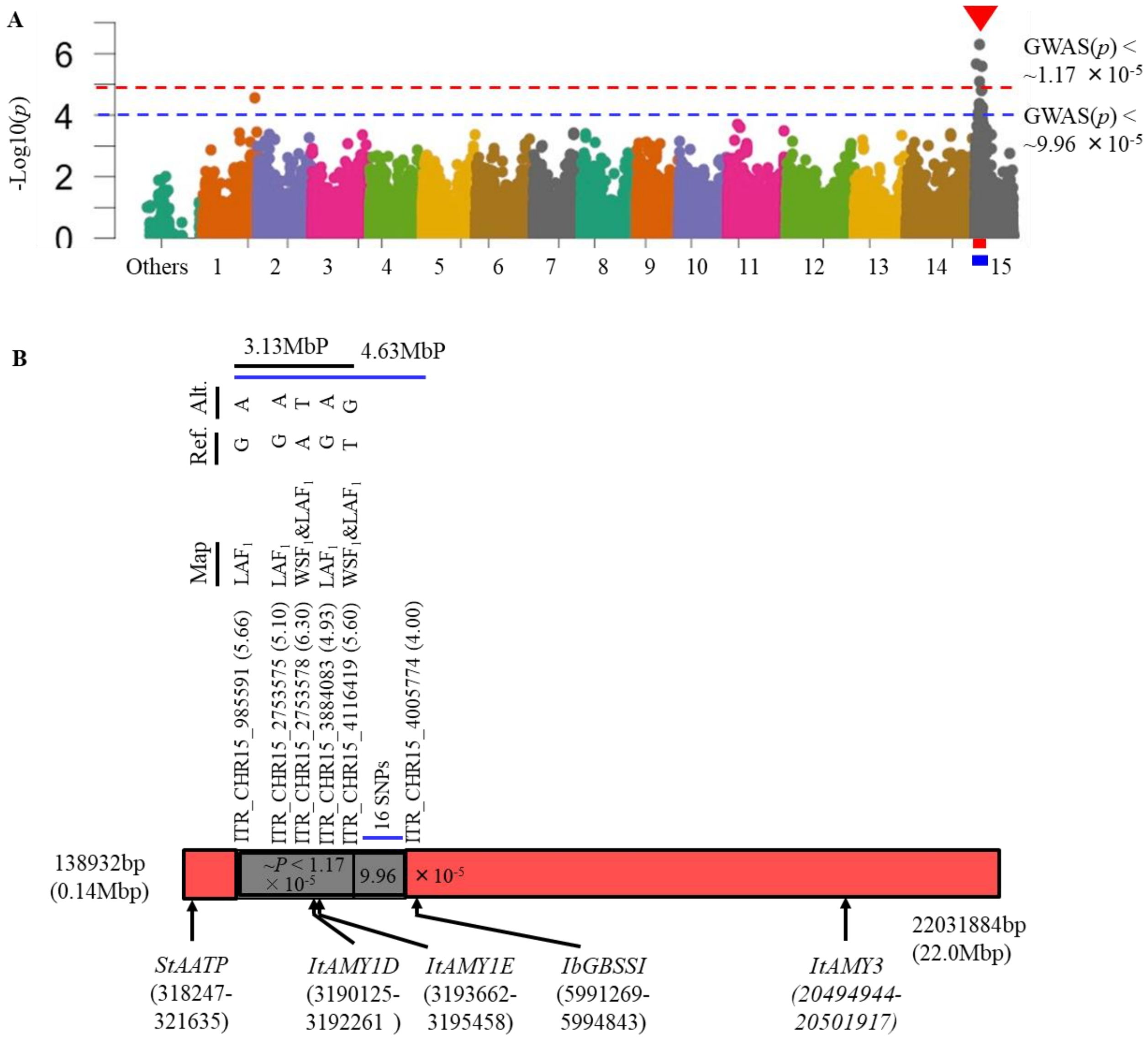

Supplementary Figure 7. Positions and covering regions by the candidate SNPs and candidate genes associated with starch metabolism on the most stable peak on HG15. **(A)** Manhattan plots for SC (LAF_1 population from 2019) showing the novel SNP loci on HG 15. The horizontal dashed red line represents the significance threshold for suggestive *P*-value value of 1.17×10^{-5}. Also an threshold line with blue color was shown for *P*-value value of 9.96×10^{-5}. **(B)** Genomic locations of five SNPs and the candidate genes associated with starch metabolism on HG 15. The –log10(*P*) for each SNP is shown in parenthesis. The allele determining types (haplotypes) of SC (KNS or AKM) in the F_1 populations were also shown. Chr, chromosome; Ref, reference; Alt, alternative. WSF_1, whole set F_1 population; LAF_1, low anthocyanin containing F_1 population.

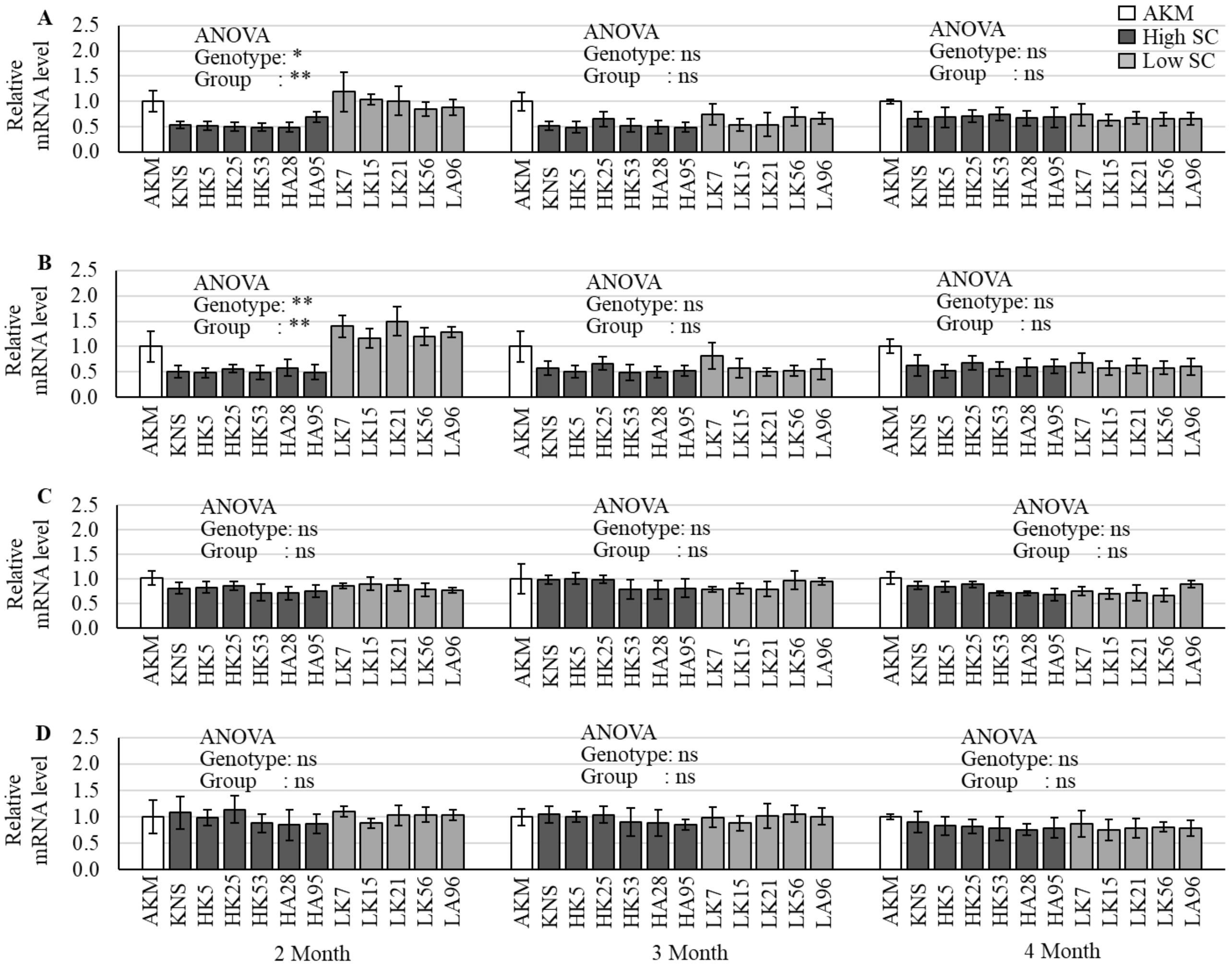

Supplementary Figure 8. Real-time PCR of starch metabolizing genes in the storage roots of AKM, KNS, five high and five low starch containing lines at 2, 3 and 4 months after field transplantation. **(A), (B)**, **(C)** and **(D)** are the expression of *IbAMY1D, IbAMY1E, IbAMY3* and *IbAATP*, respectively. Gene expressions are presented relative to the expression level of AKM plants. The error bars represent the standard error of the measurement for 3 independent biological replications (*n* =3). Transcript levels were normalized to an *IbActin* gene as an internal control. No asterisks above the bars indicate ΔCt values [= Ct(*target*) − Ct (*Actin*)] that are not significantly different from those of the AKM plants, as revealed by pair-wise *t* test. For analysis of variance (ANOVA) among 12 genotypes or 3 groups (AKM, high SC and low SC), ns, single and double asterisks indicate the statistical significance of the differences of non-significant, $P < 0.05$ and $P < 0.01$, respectively. H, high starch containing F_1 lines; L, high starch containing F_1 lines.
